# Optimizing the Steering of Driverless Personal Mobility Pods with a Novel Differential Harris Hawks Optimization Algorithm (DHHO) and Encoder Modeling

**DOI:** 10.3390/s24144650

**Published:** 2024-07-17

**Authors:** Mohamed Reda, Ahmed Onsy, Amira Y. Haikal, Ali Ghanbari

**Affiliations:** 1 School of Engineering, University of Central Lancashire, Preston PR1 2HE, UK; 2 Computers and Control Systems Engineering Department, Faculty of Engineering, Mansoura University, Mansoura 35516, Egypt

**Keywords:** steering control, steering angle encoder, driverless pod, Ackermann steering, electric power steering, Harris Hawks optimization, CEC2020 benchmark, transient response

## Abstract

This paper aims to improve the steering performance of the Ackermann personal mobility scooter based on a new meta-heuristic optimization algorithm named Differential Harris Hawks Optimization (DHHO) and the modeling of the steering encoder. The steering response in the Ackermann mechanism is crucial for automated driving systems (ADS), especially in localization and path-planning phases. Various methods presented in the literature are used to control the steering, and meta-heuristic optimization algorithms have achieved prominent results. Harris Hawks optimization (HHO) algorithm is a recent algorithm that outperforms state-of-the-art algorithms in various optimization applications. However, it has yet to be applied to the steering control application. The research in this paper was conducted in three stages. First, practical experiments were performed on the steering encoder sensor that measures the steering angle of the Landlex mobility scooter, and supervised learning was applied to model the results obtained for the steering control. Second, the DHHO algorithm is proposed by introducing mutation between hawks in the exploration phase instead of the Hawks perch technique, improving population diversity and reducing premature convergence. The simulation results on CEC2021 benchmark functions showed that the DHHO algorithm outperforms the HHO, PSO, BAS, and CMAES algorithms. The mean error of the DHHO is improved with a confidence level of 99.8047% and 91.6016% in the 10-dimension and 20-dimension problems, respectively, compared with the original HHO. Third, DHHO is implemented for interactive real-time PID tuning to control the steering of the Ackermann scooter. The practical transient response results showed that the settling time is improved by 89.31% compared to the original response with no overshoot and steady-state error, proving the superior performance of the DHHO algorithm compared to the traditional control methods.

## 1. Introduction

The steering mechanism has multiple types based on the number of wheels and the overall layout of the vehicle. Two-wheel differential drive with passive cater(s) typically consists of two powered wheels with one or more passive cater(s) for stability [1]. The two-wheel differential drive with one powered steering caster is similar to the previous one, except there is a powered steering caster for direction control [2]. Four-wheel differential drive (skid steering) is a mechanism where varying wheel speeds enable steering. The car has four motors connected to the four wheels, enabling speed control on each wheel [3]. Rear-wheel forklift steering is a mechanism where the steering is carried out primarily via the rear wheels, and is widely used in warehouse forklifts [4].

An all-wheel steering mechanism is a mechanism where all wheels can independently steer, allowing a high degree of mobility and maneuvering [5]. Rocker Bogie mechanism is a system where the chassis’s pitch is averaged and altered to maintain weight distribution and wheel–ground contact [6]. Omni-wheel (Mecanum) steering utilizes omni-wheels for multi-direction movements, enabling movement in diagonal directions [7]. Crab steering is a four-wheel drive mechanism that allows horizontal translation, and all wheels can turn in four-point turns [8].

Ackerman’s steering mechanism is the most traditional car steering mechanism used in commercial cars. It has fixed rear wheels and a front steering wheel to control the direction. It is a straightforward control geometry with non-slipping wheel turns, but it requires accurate steering mapping to determine the car location precisely [9]. This paper will study the Ackermann mechanism for a mobility scooter.

The mobility scooter prototype is based on the Ackermann mechanism, where the car’s direction and the power steering mechanism are related. The Ackermann steering architecture is a conventional steering system used in most commercial vehicles and trucks. Its main principle is based on the fact that the inside wheel travels a shorter radius than the outside wheel. The Ackermann mechanism is designed to turn the inner wheel at a greater angle than the outer wheel, leading to the inner wheel traveling a shorter radius than the outer one. In practice, when the steering wheel, the steering motor, rotates, it translates the rotation angle through the tie rods used to pull or push the steering arms, causing the wheels to turn at different angles [10].

Ackermann steering is preferred in most passenger cars due to its simplicity and efficiency. It has fewer control parameters because it only controls the steering motor fixed on the steering mechanism rather than controlling four different motor speeds. However, it provides less maneuverability in too-tight turns compared to the four-wheel differential drives. Therefore, Ackermann is preferred in passenger vehicles because the city streets are designed to fit large cars with acceptable turns [11].

The steering control is crucial in autonomous driving systems (ADS) because it helps to determine the car’s current location in the localization phase and is used in the path planning stage in the ADS. The ADS vehicles are electric-based vehicles that witnessed significant advancements in the last decade owing to their low-carbon benefits and high energy efficiency compared to traditional vehicles [12]. Therefore, the overall performance of the whole ADS in the Ackermann mechanisms relies heavily on the steering angle accuracy [13]. The steering control process involves implementing two layers: hardware represented in the controller, software that includes the control algorithm, and the firmware of the whole system.

The controller is the brain of the robot, and it is responsible for controlling all the operations of any autonomous system. The microcontroller is a computer on a single chip, sometimes named a microcomputer. There are various types of microcontrollers produced by different vendors, such as Programmable logic controllers (PLCs) for industrial applications [14], PIC microcontrollers from Microchip Technology (Chandler, AZ, USA), ARM microcontrollers from ARM Holdings (Cambridge, UK) [15], myRIO from National Instrument (Austin, TX, USA) [16], AVR from Atmel (now part of Microchip Technology, Chandler, AZ, USA) which is the core of the Arduinos [17], Jetson from NVIDIA (Santa Clara, CA, USA) [18], and Raspberry Pi from the Raspberry Pi Foundation (Cambridge, UK) [19].

The control algorithm represents the methods to minimize errors between the desired and actual steering angles in automatic closed-loop control systems. These control algorithms can be implemented as code compiled on the controller. There are various control algorithms: classic PID controllers and Intelligent controllers. Classic PID controllers are used worldwide in more than 95% of control system applications. It is simple, easy to implement, and highly effective [20].

PID controllers are based on three components: proportional term, integral term, and derivative time. The main disadvantage of the PID controller is the time-consuming parameter tuning process. Multiple methods are used for parameter tuning, such as the traditional methods, Ziegler–Nichols (ZN), and intelligent meta-heuristic optimization algorithms, such as the PSO algorithm.

Multiple meta-heuristic optimization algorithms, such as PSO, CMAES, and BAS algorithms, have been used for PID tuning in steering control. One of the most recent algorithms is the Harris Hawks optimization (HHO) algorithm, introduced in 2019. This algorithm proved its efficiency in multiple applications, including PID tuning, which outperforms state-of-the-art algorithms [21]. However, to the author’s best knowledge, the HHO algorithm has yet to be applied to the PID tuning for steering control. Despite the promising results of the HHO algorithm, the HHO lacks diversity in its population, which requires improvements [22]. Therefore, the proposed algorithm is based on a meta-heuristic optimization algorithm for parameter tuning.

The main contributions of this paper are summarized as follows:

- A novel variant called the Differential Harris Hawks Optimization (DHHO) algorithm is introduced to improve the premature convergence and the population diversity of the traditional HHO algorithm.
- Practical experiments are conducted on the steering encoder sensor in the power steering mechanism, and supervised learning is applied to deduce the steering model of the sensor necessary for the steering control.
- Practical implementation of the closed-loop steering control system on the Landlex Broadway RS (Landlex, Manchester, UK) personal mobility scooter.
- The proposed DHHO algorithm is applied and validated for real-time PID tuning for the steering control of the scooter.
- Simulation results: The DHHO algorithm improved the mean error by 99.8047% and 91.6016% in the 10-dimension and 20-dimension of the CEC2021 benchmark functions compared with the traditional HHO algorithm.
- Practical results: The transient response results of the steering control showed that the settling time is improved by 89.31% compared with the original response with no overshoot and settling time.

The rest of the paper is organized as follows: Section 2 discusses the previous research performed on the scooter and its drawbacks. The state-of-the-art algorithms used for PID tuning and the standard HHO algorithm are also discussed. Section 3 introduces the proposed DHHO optimization algorithm. Section 4 presents the diversity analysis between the DHHO and the HHO algorithm as a proof of concept. Section 5 illustrates the validation and the simulation results of the proposed DHHO and the state-of-the-art algorithms on CEC2020/2021 benchmark functions. Section 6 presents the hardware contributions to the scooter, including the system’s hardware modifications and practical experiments on the steering mechanism to draw the relationships needed for the steering control. Section 7 explains the practical application of the proposed DHHO algorithm to the real-time PID tuning in the steering control and the transient response results. Section 8 presents an overall discussion of the results for all the experiments conducted in this paper. Finally, the summary and directions for future work are presented in Section 9.

## 2. Related Work

The related work is explained in three segments: First, the Landlex personal mobility scooter is described, and modifications are made to it in the related work. Second, the state-of-the-art PID tuning algorithms in the steering control are discussed. Finally, the standard HHO algorithm is explained in detail as a preliminary step before explaining the methodology.

### 2.1. Landlex Broadway RS Personal Mobility Scooter

Commercial mobility scooters, used by individuals with limited mobility, are excellent examples of vehicles deploying the Ackermann steering mechanism. This mechanism is essential in these scooters to guarantee smooth and secure navigation. The typical mobility scooter used in this study is the Landlex Broadway RS, the four-wheel model S400xR-RS [23]. It implements the Ackermann steering system using a straightforward linkage setup connected to the front wheels.

**Figure 1:**
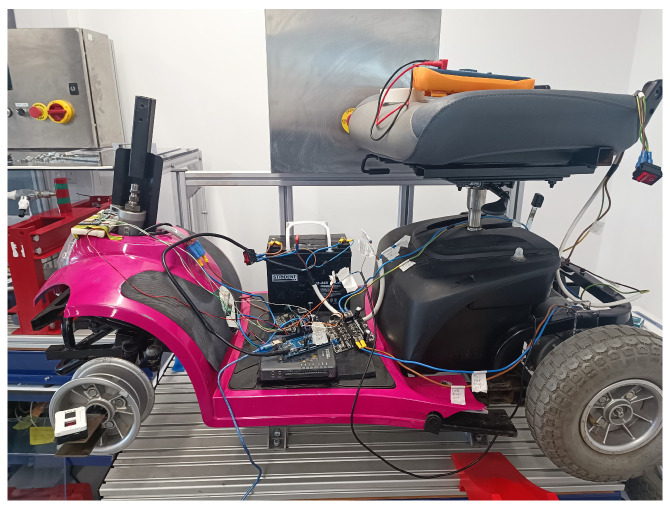
Landlex Broadway RS, the 4-wheel model S400xR-RS Mobility Scooter.

Philip et al. proposed a modified version of the Landlex Broadway RS Scooter. The scooter was modified to make it driverless by adding an electric-powered steering motor from a Vauxhall Corsa C (Vauxhall Motors, Luton, UK) and replacing the manual controller with an NI myRio controller to control the scooter [24]. The scooter layout is shown in Figure 1.

The scooter in [24] has a set of drawbacks, and multiple points need to be improved for better performance. First, the power steering motor used to control the steering angle is a geared DC motor, in which the performance of the steering response is strongly related to the PWM signal applied to the DC motor. Applying a high PWM signal for a slight steering angle will make the steering mechanisms jerky and cause sudden turns, which is undesirable. Therefore, adaptive steering control is required to handle this problem.

Second, the angular throttle potentiometer (encoder) is not calibrated, and there is no relationship between the steering motor angle and the encoder angle. Therefore, the encoder signal will be meaningless when it is fed back to the controller because the corresponding angle of the steering motor is unknown, leading to inaccurate steering control; it acts as an open-loop controller. Therefore, the relationship between the potentiometer reading and the steering motor angle must be obtained.

In addition, the Ackermann mechanism in the Landlex Broadway RS Scooter is different from the exact standard Ackerman. Hence, the mechanism’s geometry does not accurately represent the steering angle for the front wheels. Moreover, mounting the power steering motor adds unmeasurable left and right angle variations. This problem will lead to wrong steering and wrong destination for the path planning. Therefore, the relationship between the motor angle and the left and the right wheel steering angles must be obtained.

Furthermore, the myRio-1900 controller (National Instrument, Austin, TX, USA) has a powerful processor, but its operating system is NI Linux Real-time (part of LabVIEW 2021), which is not a pure Linux operating system [25]. Installing an ROS-based system on NI Linux is not straightforward and requires dependencies unavailable in the NI feeds of the myRio-1900 [26]. Therefore, the myRio-1900 controller must be replaced by a Linux-based controller, which supports ROS dependencies. Raspberry Pi controller is a suitable choice for the proposed system.

### 2.2. PID Tuning Algorithms

A PID controller controls the steering angle of the mobility scooter. It requires parameter tuning to find the relevant gain values that are hardware-dependent. Traditional techniques, such as manual tuning and Ziegler–Nichols (ZN) techniques, can be used but do not provide accurate results. However, they can be used to give an appropriate starting point [27]. Meta-heuristic optimization algorithms are widely used to solve various optimization problems. Recently, they are applied to the PID controller to obtain the parameter values of the PID controller.

Zhou et al. introduced a PID controller to control the steering of an automated guided vehicle (AVG) optimized by particle swarm optimization (PSO) algorithm, which significantly improved the performance [28]. Gao et al. proposed a PID controller tuned based on the PSO algorithm to control the vehicle’s speed, which improved convergence speed [29]. Maki et al. proposed a PD controller tuned by the Covariance Matrix Adaption Evolution Strategy (CMA-ES) algorithm to improve the steering performance, which improved the performance compared to the traditional methods [30].

Zhang et al. introduced a PID controller to control the steering angle of an Ackerman-based Automated Guided vehicle based on the Beetle Antennae Search (BAS) algorithm, outperforming the traditional PID controller [31]. Moshayedi et al. applied different algorithms for the PID tuning to control AGV’s steering, including Ziegler–Nichols (ZN), PSO algorithm, and BAS algorithms. Results of the ZN, PSO, and BAS algorithms provided error values close to each other. The fastest algorithm was the BAS, and the best performance was the PSO algorithm [32]. He et al. proposed a PI controller for steering control in self-driving vehicles (especially trucks) based on the PSO algorithm, which results in stable final error [33]. Zhang et al. proposed a PSO-based PID controller to control the steering of an unmanned ground vehicle, which provided a feasible performance [34].

One of the most recent meta-heuristic algorithms is the Harris Hawks Optimization (HHO) algorithm. This algorithm was first introduced by Heidari et al. in 2019, and it outperformed robust algorithms such as PSO, GA, GWO, FA, DE, and more [35]. This algorithm has been applied to various applications theoretically and practically [36]. HHO has been used for the PID parameter tuning problem for many applications, such as speed control and multiple electrical applications. Ekinci et al. proposed an HHO-based PID controller to improve the performance and stability of the automatic voltage regulator (AVR) system, outperforming the BBO algorithm [37]. Elkady et al. proposed an HHO-PI controller to improve the transient response of the dynamic voltage restorer (DVR) system, outperforming the PSO and WOA algorithms [38].

Ekinci et al. proposed another HHO-PID controller for regulating the speed of a DC motor, outperforming ASO, GWO, and SCA algorithms [39]. Izci et al. proposed a PID controller optimized by the HHO to control the aircraft pitch angle, outperforming SSA and ASO algorithms [40]. Munagala et al. presented an HHO-PID controller to control the speed of a DC motor, outperforming GWO and SFS algorithms [41]. Sahu et al. proposed an HHO-based PID controller to control a solar-thermal system’s load frequency (LFC), outperforming the traditional controller [42]. Karnavas et al. presented an HHO-PID controller to control the frequency of power generation systems, outperforming the PSO-based controller [43].

Izci et al. introduced an HHO-based PID controller to control the terminal voltage in the automatic voltage regulator (AVR) system, outperforming the traditional PID controller in terms of transient response [44]. Rani et al. proposed an HHO-PI controller to control the harmonics on the grid side of the voltage source converter (VSC), which was successfully verified and compared with the other converters [45]. Aldin et al. presented an HHO-PI controller to control the speed of the permanent magnet synchronous wind generator (PMSWG) system, outperforming the traditional PID and adaptive fuzzy logic (AFLC) controllers [46].

While various optimization algorithms have been successfully applied to PID tuning for steering control, each has its limitations. The CMA-ES algorithm can suffer from high computational complexity, making it less efficient for real-time applications than the PSO, BAS, and HHO algorithms. The PSO algorithm is effective in PID tuning due to its simplicity and low time complexity. However, PSO may converge prematurely to local optima because it lacks separate exploration and exploitation phases and is parameter-dependent, leading to an imbalance between local search (exploitation) and global search (exploration) [47]. The BAS algorithm is considered the fastest compared to PSO, HHO, and CMA-ES, but it lacks robustness in diverse optimization landscapes, resulting in lower-quality solutions [32]. Although the HHO algorithm has shown success in PID tuning for various applications, it has not yet been applied to the PID tuning of steering control applications. The primary limitation of the original HHO algorithm is its low population diversity, which can result in the exclusion of valuable search spaces [22].

The proposed Differential Harris Hawks Optimization (DHHO) algorithm addresses these issues by introducing a Hawks mutation operator in the exploration phase, enhancing diversity and global search capabilities and thereby improving performance in complex optimization scenarios such as steering control. The DHHO algorithm is distinguished by having a separate stage for exploration and a separate stage for exploitation, both controlled by a dynamic parameter called the escaping energy, similar to HHO. This adaptive parameter balances the two phases and overcomes the single-stage and parameter-dependency issues in the PSO algorithm. The DHHO algorithm is also less complex than the CMA-ES algorithm, resulting in lower computational time. The enhanced diversity is expected to reduce the chances of getting trapped in local minima and outperform the original HHO, PSO, BAS, and CMA-ES algorithms, which will be proved in the diversity analysis and benchmark testing.

### 2.3. Standard Harris Hawks Optimization (HHO) Algorithm

The cooperative hunting behavior of the Harris hawks in nature inspires the HHO algorithm. The population’s candidate solutions are called Hawks, predators that hunt for prey. The prey called the rabbit in the HHO algorithm represents the best solution for the population. The objective is to find the best prey (rabbit) to be haunted by the hawks. During the iterations, the hawks evolved via the exploration and exploitation process to find the rabbit. This algorithm mathematically relies on three main components: the escaping energy, the exploitation based on the besiege and leapfrog technique, and the exploration based on the Harris’ hawks perch method. The algorithm is based on the dynamic transition between exploration and exploitation based on the escaping energy of the prey (the solution). This dynamic behavior between global and local search provides the algorithm with powerful search capability [35].

The first component is the escaping energy (E), which is vital in switching between the exploration and exportation phases. Therefore, it decides how the population’s solutions are updated. The high energy at the beginning switches the exploration phase, which leads to tremendous and random changes in the hawk. This exploration process represents the search for prey. As the algorithm iteration increases, the energy becomes low, which switches the exploitation phase, which means the hawks are updated to move closer to the best solution (the rabbit). This exploitation process mimics the procedure of attacking the prey. The escaping energy *E* is calculated from Equation (1), where E0 is a randomly generated factor to introduce variability in the escaping energy, and E1 is a decreasing energy factor that is inversely proportional to the ratio between the current iteration *t* and the maximum iterations *T*. *E* represents the decreasing energy of the prey as it tries to escape from the hawks.

(1) $$E=E_{1}\cdot E_{0}\;\mathrm{where}\;\left\{\begin{array}{cc} E_{1} & =2\cdot \left(1-\frac{t}{T}\right)\\ E_{0} & =2\cdot \mathrm{rand}()-1 \end{array}\right.$$

The exploration phase in the HHO algorithm represents the hawks’ searching the environments to locate the prey. The main objective of this process is to explore most of the search space. The exploration phase aims to give the population high diversity to avoid falling into a local minimum. In the HHO, the exploration is based on random movements by the hawks based on the positions of other hawks in the population or randomly selected locations, as defined in Equation (2). A random number *q* between 0 and 1 is generated; if it is less than 0.5, the new hawk’s position $X_{new}$ is generated based on the current hawk’s position *X*, and a randomly selected hawk $X_{rand}$. If *q* is greater than or equal to 0.5, $X_{new}$ is generated based on the mean positions of the hawks $X_{mean}$, and the rabbit *R* that represents the best solution. The lower and upper bounds are *U* and *L*, and $r_{3},r_{4},r_{5},r_{6}$ are random numbers between 0 and 1.

(2) $$X_{\mathrm{new}}=\left\{\begin{array}{cc} X_{\mathrm{rand}}-r_{3}\left|X_{\mathrm{rand}}-2r_{4}X\right| & \mathrm{if}\;q<0.5\\ \left(R-X_{\mathrm{Mean}}\right)-r_{5}\left(L+r_{6}\left(U-L\right)\right) & \mathrm{if}\;q\geq 0.5 \end{array}\right.$$

The exploitation process in the HHO algorithm represents a strategic approach by the hawks to catch the spotted prey. This process is mathematically described by a set of cases as shown in Equation (3). This process involves using the best current prey (rabbit) *R* to guide the search. It consists of four techniques based on the rabbit energy *E* and the jumping strength of the rabbit *J*, where J=2·(1−rand()). The four techniques are the soft besiege (deceptive approach), the hard besiege (direct attack), and the leapfrog movements in two phases. Where $r,r_{1},$ and $r_{2}$ are random numbers between 0 and 1, and Levy() is a function introducing random walks (called Levy flights) for diversification.

(3) $$X_{\mathrm{new}}=\left\{\begin{array}{cc} R-E\cdot |R-X|, & \mathrm{if}\;r\geq 0.5\;\mathrm{and}\;|E|<0.5\\ (R-X)-E\cdot |J\cdot R-X|, & \mathrm{if}\;r\geq 0.5\;\mathrm{and}\;|E|\geq 0.5\\ R-E\cdot |J\cdot R-X|\;+\;r_{1}\cdot \mathrm{Levy}\left(\right), & \mathrm{if}\;r<0.5\;\mathrm{and}\;|E|\geq 0.5\\ R-E\cdot |J\cdot R\;-\;X_{\mathrm{Mean}}|\;+\;r_{2}\cdot \mathrm{Levy}\left(\right), & \mathrm{if}\;r<0.5\;\mathrm{and}\;|E|<0.5 \end{array}\right.$$

The algorithm starts by initializing a random population of candidate solutions called Hawks. Second, the algorithm defines the best solution in the population as a rabbit *R* based on the fitness function. Next, the algorithm starts the main loop and continues until it reaches the maximum number of iterations. Third, the energy *E* is updated from Equation (1); if the energy is high, then the exploration equation Equation (2) is applied to update the hawk. If the energy is low, then the exploitation equation Equation (3) is applied to update the hawk. Fourth, the fitness of the updated hawk is calculated, and the rabbit is updated if the new hawk is more fit than the rabbit. At the end of the algorithm, the best solution is the rabbit *R*. The pseudocode of the original HHO algorithm is presented in the Supplementary Materials File (Algorithm S1).

## 3. The Proposed Differential Harris Hawks Optimization (DHHO) Algorithm

The main drawback of the original HHO algorithm is its susceptibility to becoming trapped into the local minimum due to the low population diversity, leading to missing some valuable search spaces [22]. This low diversity leads to premature convergence of the search process [48]. The structure of the HHO algorithm allows the hybridization of different exploration strategies that can improve the diversity of the population and reduce the possibility of getting trapped in a local minimum [21].

The proposed DHHO algorithm is based on introducing reproduction and replication between hawks to generate a diverse population. The main objective of the algorithm is to improve the diversity of the Hawks’ population during the exploration phase. The DHHO algorithm enhances the exploration capability by deploying the mutation operator DE/rand/1 as an exploration method instead of Harris’ Hawks perch technique in Equation (2).

The DHHO algorithm starts by initializing a random population *P* consisting of *N* hawks; each hawk $X_{i}$ represents a candidate solution for the optimization problem. The hawk consists of *D* dimensions, where $X_{i,j}$ is the *j*-th dimension of the *i*-th hawk. The population set *P* can be formulated as in Equation (4).

(4) $$X=\{X_{i,j}|i=1,2,\ldots ,N;\;j=1,2,\ldots ,D\}$$

The fitness function f(X) is used to evaluate the quality of the solution *X* (hawk) for the optimization problem. The fitness function mainly represents an error function, so a low fineness value means low error, which shows a high-quality solution. On the other hand, a high fitness value means a low-quality solution. The global best represents the solution with the minimum fitness value, which represents the final solution of the optimization problem, and it is called the Rabbit *R*. At the beginning of the algorithm, the quality of the solutions in the initial population is low due to the random initialization. As the algorithm proceeds, the quality of the solutions improves.

The loop of the algorithm starts by iterating each hawk $X_{i}$ in the population *X*. First, the escaping energy *E* is calculated to the current hawk $X_{i}$ using Equation (1), as in the standard HHO algorithm. The escaping energy *E* switches between two primary phases: exploration and exploitation. If *E* is low (less than 1), the hawk $X_{i}$ undergoes the exploitation phase using the third and fourth cases in Equation (3), as in the standard HHO algorithm.

On the other hand, if *E* is high (more than 1), the hawk $X_{i}$ undergoes the exploration phase, which is modified in the DHHO algorithm; Equation (2) in the standard HHO algorithm is replaced by the mutation operator DE/rand/1. In this operator, the position of the mutant hawk $V_{i}^{g}$ is randomly generated based on three randomly selected hawks from the population $X_{r1}^{g},X_{r2}^{g},$ and $X_{r3}^{g}$, as in Equation (5). The scaling mutation factor *F* is set to 0.5. The mutant hawk $V_{i}^{g}$ represents a new hawk with different genes generated by the mixture of three random hawks, indicating a diverse hawk.

(5) $$V_{i}^{g}=X_{r1}^{g}+F\cdot \left(X_{r2}^{g}-X_{r3}^{g}\right)$$

A binomial (uniform) crossover operator is applied between the mutant hawk $V_{i}^{g}$ and the current hawk $X_{i}^{g}$ according to Equation (6). The mutant hawk and the current hawk represent the parents of the newly generated child hawk $U_{i}^{g}$. Each dimension of the child hawk $U_{i,j}^{g}$ selected between the two corresponding parents’ dimensions based on a crossover probability CR, which is set to 0.5 (50%) to give an equal chance for the two parents. The child hawk $U_{i}^{g}$ is generated based on the replication between two parents, and the child carries 50% of the genes from the first parent $X_{i}^{g}$ (the current hawk) and 50% from the other parent $V_{i}^{g}$ (the mutant hawk).

Finally, the new hawk $X_{i}^{g+1}$ is selected between the child hawk $U_{i}^{g}$ and the current hawk $X_{i}^{g}$ based on the survival of the fittest principle, as in Equation (7). The fitness of child hawk $f(U_{i}^{g})$ is calculated and is compared with the fitness of the current hawk $f(X_{i}^{g})$. If the child hawk has better fitness than the current hawk, the child hawk $U_{i}^{g}$ will pass to the next generation. On the other hand, if the current hawk is better than the child hawk, the parent (current) hawk $X_{i}^{g}$ will pass to the next generation.

(6) $$U_{i,j}^{g}=\left\{\begin{array}{cc} V_{i,j}^{g} & \mathrm{if}\;{\mathrm{rand}}_{i,j}\leq CR\\ X_{i,j}^{g} & \mathrm{otherwise} \end{array}\right.$$

(7) $$X_{i}^{g+1}=\left\{\begin{array}{cc} U_{i}^{g} & \mathrm{if}\;(f\left(U_{i}^{g}\right)\leq f\left(X_{i}^{g}\right))\\ X_{i}^{g} & \mathrm{otherwise} \end{array}\right.$$

Algorithm 1 shows the pseudocode of the steps of the DHHO algorithm. The algorithm starts by initializing a random population of candidate solutions called Hawks. Second, the algorithm defines the best solution in the population as a rabbit *R* based on the fitness function.

Next, the algorithm starts the main loop and continues until it reaches the maximum number of iterations. Third, the energy *E* is updated from Equation (1), as this energy determines how the hawk will be updated. If the energy is high, then the modified exploration equation Equation (5) is applied to update the hawk. Next, the crossover and selection operator are applied using Equation (6) and Equation (7), respectively. These equations ensure a higher diversity of the population than the one from the traditional HHO exploration in Equation (2).

If the energy is low, then the third and fourth cases of the exploitation equation Equation (3) is applied to update the hawk. Fourth, the fitness of the updated hawk is calculated, and the rabbit is updated if the new hawk is more fit than the rabbit. At the end of the algorithm, the best solution is the rabbit *R*. A flowchart of the DHHO algorithm is presented in the Supplementary Materials File (Figure S1).
**Algorithm 1** Differential Harris Hawks Optimization (DHHO) Algorithm  1:Initialize the population $X_{i}$, i=1,2,…,N  2:Evaluate the fitness of each hawk  3:Initialize the best solution found so far as the rabbit’s location *R*  4:**for** t=1 to *T* **do**                                                 ▹ T is the maximum number of iterations  5:      **for** each hawk *i* **do**  6:            Update energy *E* of the rabbit from Equation (1)  7:            **if** Rabbit’s energy is high **then**                                                     ▹ Exploration phase  8:                 Generate mutant hawks using Equation (5).  9:                 Apply crossover between the current and the mutant hawks using Equation (6).10:                Apply selection method to get the new hawk’s position using Equation (7).11:           **else**                                                                                                 ▹ Exploitation phase12:                Update hawk’s position based on the third and fourth cases of Equation (3)13:           **end if**14:           Evaluate fitness of hawk *i*15:           **if** Fitness of hawk *i* is better than *R* **then**16:                Update *R* with hawk *i*’s position17:           **end if**18:     **end for**19:     Record the best solution found so far20:**end for**21:**return** Best solution found *R*

## 4. Exploration and Diversity Analysis (HHO vs. DHHO) as a Proof of Concept

The main objective of the DHHO algorithm is to improve the diversity and exploration capability of the original HHO algorithm. This section conducts a fair experiment between the HHO and DHHO algorithms to evaluate both populations’ diversity. The exploration method of the DHHO algorithm involves the mutation operator as in Equation (5). At the same time, the exploration technique of the HHO algorithm is based on perching and random tall trees as in Equation (2). The assumed hypothesis that the DHHO has more diversity than the HHO will be proved through statistical analysis of multiple dimensions search spaces. First, both algorithms started with the same initial random population of 30 individuals.

The experiment is performed on multiple dimensions, varying between 10 and 100, with a step of 10. The lower and upper ranges are assumed to be between −10 and 10 for all dimensions of both algorithms. The termination condition is set to a maximum number of iterations of 300. Due to the existence of some random parameters, each experiment for each dimension was performed for 50 individual runs. Then, the average performance is calculated for all the runs.

The experiment only focuses on measuring the average variance and the standard deviation in the dimensions of each individual in the population throughout the iterations and runs. This method stresses the population’s diversity rather than the algorithm’s convergence. High variance means high diversity in the population, resulting in a diverse population with less possibility of falling into a local minimum, and this is the main objective of the exploration process.

**Figure 2:**
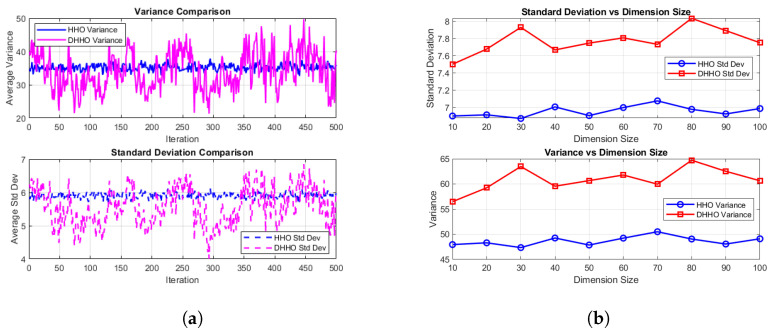
Diversity plots between HHO and DHHO exploration methods. (**a**) Mean diversity plots (variance and SD). (**b**) Dimensions size vs. Variance.

The average variance of all 50 runs among all the individuals has been stored and monitored over the iteration progress. Figure 2a displays the mean-variance and the mean, standard deviation for the two exploration methods for all the dimensions. The detailed diversity plots for each dimension from 10 to 100 are presented in the Supplementary Materials File (Figure S2).

Table 1 shows the results for each exploration method for all the dimensions, where the first column represents the size of the dimensions, the second column is the algorithm name, the third column in the average peak variance for all the individuals in the population over the 50 runs, and the fourth column is the standard deviation for them.

Analyzing the variance and the standard deviation (SD), it is evident that the DHHO algorithm exhibits a higher variance and SD than the HHO algorithm for all the dimensions. This finding suggests that the DHHO can explore a broader search space range, indicating greater population diversity during the exploration phase. As the dimensions increase, both algorithms’ variance and SD increase, but the increase rate of DHHO is much more than the HHO, as seen in Figure 2b.

The higher diversity of the exploration method proposed in the DHHO shows its ability to cover a broader range of the search space. It is less likely to become trapped in local optima and reduces the risk of premature convergence. It also has better chances of finding a global optimum while exploring more solutions.

The Wilcoxon signed-rank test is conducted for both the variance and the SD across different dimension sizes to prove the significance of the results. Regarding the variance comparison, the *p*-value is 0.001953125, less than the alpha level of 0.05, indicating the significance of the DHHO exploration compared to the HHO exploration. Concerning the SD metric, the *p*-value also equals 0.001953125, which is less than 0.05, indicating the significant advantage of the DHHO exploration capability over the HHO.

In summary, based on the statistical analysis and the observed results, the DHHO algorithm’s exploration demonstrates a superior ability to explore more search spaces compared to the HHO algorithm. Therefore, replacing the HHO exploration technique with the mutation operator in the DHHO algorithm leads to a more robust algorithm for solving complex optimization algorithms.

## 5. Benchmark Testing on CEC2020/2021

This section evaluates the proposed DHHO algorithm against the meta-heuristic optimization algorithm recently used in the literature for PID tuning, as mentioned in the literature review. The DHHO algorithm is compared with the Particle swarm optimization (PSO), Covariance matrix adaptation evolution strategy (CMA-ES), Beetle Antennae Search (BAS), and original Harris Hawks Optimization (HHO) algorithms. All these algorithms will be validated for the ten benchmark functions of CEC2020/2021 for 10 and 20 dimensions.

### 5.1. Parameters Setting

Five algorithms have been compared: PSO, CMA-ES, BAS, HHO, and the proposed DHHO. The parameters setting for the algorithms are shown in Table 2. The population size is set to 30 for all the algorithms. All algorithms are tested on ten functions of CEC2020 [49], which have the same test suit for CEC2021 [50]. Each algorithm runs for 30 independent runs for the 10-dimension and the 20-dimension cases. The termination condition is set to a maximum function evaluation (MaxFES) of 200,000 for the 10-dimension case and 500,000 for the 20-dimension case.

The fitness function is set as the error function $E_{i}\left(x\right)=F_{i}\left(X\right)-F_{i}\left(X^{*}\right)$, where $F_{i}\left(X\right)$ is the fitness of the best-obtained solution by the algorithm for the *i*-th function, and $F_{i}\left(X^{*}\right)$ is the best-known solution for the *i*-th problem, as in [49]. The tolerance in error is set to 1.0E-08 as a termination condition. The search range for all the dimensions for all problems lies in the range [−100,100].

### 5.2. Results of the Benchmark Testing

The best error for each algorithm is collected in all 30 runs. The best, worst, median, mean, and standard deviation (SD) metrics are then calculated for each algorithm in all the runs. These results are presented in Table 3 for both the 10-dimension and the 20-dimension cases. The first column is the function label, the second column is the algorithm name, columns 3–7 are the error metrics for the 10-dimension case, and the last five are the error metrics for the 20-dimension case.

The results in that table are visualized using the box and violin plots. These plots show the distribution of the results in the 30 runs. In the box plots, small and compact boxes indicate the repeatability of the results. A red line in the middle of the box indicates the mean of the results. Violin plots are similar to the box plots, but density plots are included. The black line in the violin plot represents the mean, and the dashed red line represents the median.

**Figure 3:**
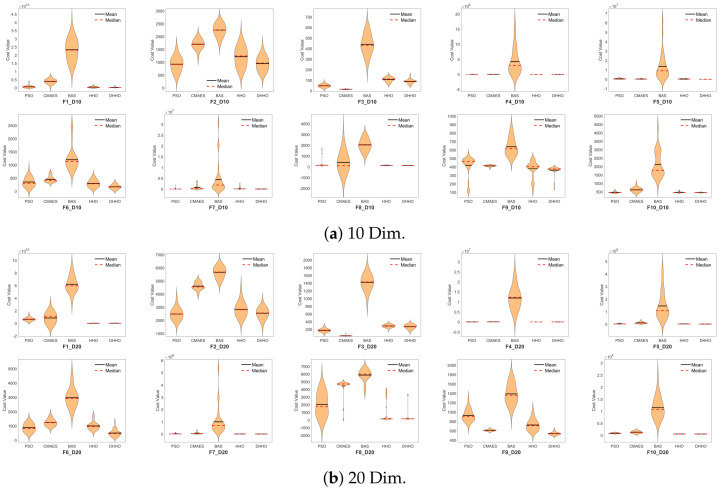
Violin plots for all the 10-Dim and 20-Dim CEC2020/2021 functions for all the algorithms in 30 runs.

Figure 3 show the violin plots for the 10-dim and the 20-dim functions. The proposed DHHO algorithm demonstrates repeatable and compact distribution of the results in most of the functions. The median and the mean results in the violin plots match the result in Table 3. On the other hand, the BAS algorithm shows the least efficient distribution compared to the other algorithms. A compact violin means more repeatability in violin plots, and a lower violin means less error. The DHHO shows the lowest and the most compact violin plots compared with the other algorithms, especially the original HHO algorithm. Visual analysis is not enough to evaluate the performance of the algorithm. Therefore, statistical analysis is performed in the following section. The box plots are similar to the violin plots but are less informative. These box plots are presented in the Supplementary Materials File (Figures S3 and S4).

### 5.3. Statistical Analysis of the Results

Group comparison is conducted using the Friedman test to obtain the ranking of the best-performing algorithms. Then, paired comparisons are held between the proposed DHHO algorithm and the other algorithms using the sign test and the Wilcoxon signed rank tests to evaluate the one-to-one performance of the algorithm. The statistical analysis is conducted on the mean and the median error results for the 10-dim and the 20-dim functions. The level of significance α is assumed to be 0.1. If the *p*-value of any test is less than the significance level, this indicates significant results. Table 4 shows the statistical analysis results in all the statistical tests used in the validation process.

The results of the Friedman test are shown in columns 4–7 in Table 4. The fourth column represents the summation of the ranks obtained by each algorithm for each function. The best algorithm with minimum error metric receives a rank value of 1, and the worst algorithm receives a rank value of 5. The fifth column is for the average rank among the ten functions for each dimension. The sixth column is the final order of the best algorithms from 1 to 5. The seventh column is the *p*-value of the Friedman test.

The *p*-value of the Friedman test in all cases is less than α, which indicates significant results. The proposed DHHO algorithm achieved first place for the mean and median metrics for the 10-Dim and the 20-Dim cases, outperforming all the other algorithms. The HHO, PSO, CMAES, and BAS algorithms achieved second, third, fourth, and fifth places, respectively. The ranking of the algorithms did not change with metrics or the dimensions.

Paired comparisons are held using the sign and Wilcoxon signed-rank tests. The DHHO is set as a reference to be compared to other algorithms. In the 10-dim case, the DHHO algorithm outperformed the HHO and the BAS algorithms in all 10 functions for the mean and median metrics. DHHO performs better than the PSO in eight functions, while PSO performs better in the other two, according to the mean and the median metrics. The DHHO algorithm has a better mean error than the CMES in nine functions and has a better median error in eight functions.

The *p*-value in the HHO case is 0.001953, which means that the DHHO algorithm is better than the original HHO algorithm by 99.8047% in the 10-dim case. In all the comparisons of the 10-dim case, the *p*-value of the Wilcoxon test is less than α, indicating the significant performance of the DHHO algorithm.

Regarding the 20-dim case, the DHHO algorithm has a better median error in 8, 9, 10, and 8 functions than the PSO, CMAES, BAS, and HHO algorithms. Similarly, the DHHO algorithm has a better mean error in 8, 9, 10, and 10 functions than the PSO, CMAES, BAS, and HHO algorithms. The *p*-value in the HHO comparison is 0.083984, which means that the DHHO is better than the original HHO algorithm by 91.6016% in the 20-dim case. In all comparisons, all the mean *p*-values of the Wilcoxon test are less than α, showing superior performance of the DHHO compared with the other algorithms. The statistical analysis proved the superiority of the proposed DHHO algorithm compared to the original HHO and the other algorithms.

**Figure 4:**
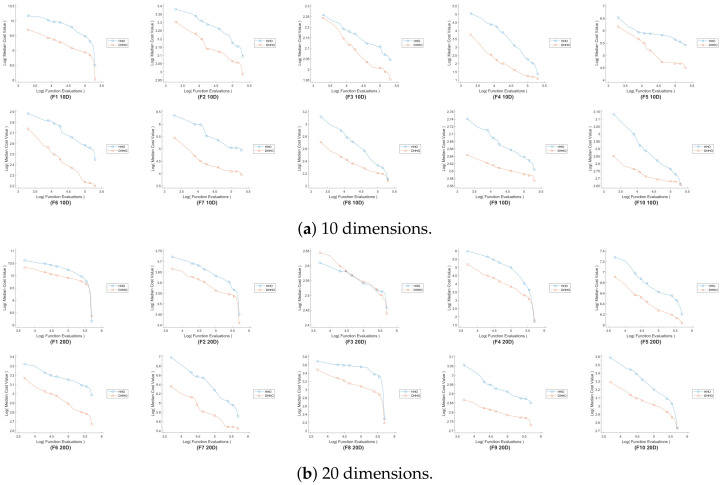
Convergence plots for median error progress in 30 runs in all the CEC2020/2021 functions for the DHHO and the original HHO algorithms.

Figure 4 shows the convergence graphs for the proposed DHHO algorithm compared with the original HHO algorithm for all the functions in the 10-dim and the 20-dim cases. These graphs represent the progress of the median error across the 30 runs, starting from the initial population until the algorithm terminates. The DHHO algorithm demonstrates better convergence than the HHO algorithm. The overall performance of the proposed DHHO algorithm outperforms the traditional HHO and the other algorithms in terms of diversity, convergence, and error. In the next step, the DHHO algorithm will be applied to the steering control problem as a practical validation for the algorithm.

## 6. Scooter Hardware Design and the Proposed Steering Angle Mapping

This section sheds light on the contributions made to the scooter hardware. Moreover, the proposed mapping experiment for the steering angle and its results are discussed.

### 6.1. Overall View of the Enhanced Scooter Prototype

**Figure 5:**
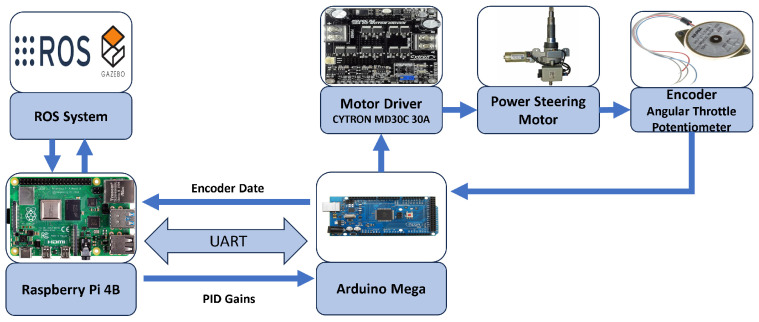
Overall hardware block diagram of the steering system of the scooter prototype.

Figure 5 shows a hardware block diagram of the enhanced scooter prototype and how the components are connected to form the steering control system. The steering system’s hardware consists of a set of hardware categories: two controllers, a steering encoder (potentiometer), and a power steering motor with a motor driver. The two controllers are the Raspberry Pi and the Arduino Mega. The rear motor and encoder can be added to the proposed system. However, the primary concern of this research is the steering control.

The power steering motor used in the scooter is the Vauxhall Corsa C (Vauxhall Motors, Luton, UK) column [54,55], which is controlled using the CYTRON MD30C 30A motor driver (Cytron Tech, Bukit Mertajam, Malaysia) that handles and supplies power to the steering motor [56]. A dedicated Strident lead acid battery of type GP12-34X (Strident Innovation, Swaffham, UK) with a capacity of 34AH supplies the steering motor through the motor driver [57]. This battery provides power to the control circuit, including the Arduino. The typical minimum PWM that can rotate the motor is 16% duty cycle. If the PWM is set to less than 16% duty cycle, the motor spins in place and does not rotate. In the enhanced scooter, the electric power steering motor does not only assist the steering. However, it fully controls the steering for the driverless scooter. Therefore, the mechanical part that connects the steering wheel with the power steering column is removed. The steering motor takes complete control of the steering.

An angular potentiometer is attached at the bottom of the power steering columns to measure the motor’s rotation angle. The potentiometer acts as a voltage divider where the voltage varies from 0 V to 5 V as the motor shaft rotates from left to right. The potentiometer output is an analog output that gives continuous voltage readings. This output is connected to an analog input in the Arduino, typically the A0 pin, that involves an analog-to-digital (ADC) conversion process inside the Arduino. The Arduino is supported by a 10-bit ADC module that maps the analog readings from 0 to 5 V are mapped to a digital reading from 0 to 1023 ($2^{10}-1$) with a resolution (sensitivity) around 4.88mV per unit (5 V/1024).

The primary controller, the Raspberry Pi Model 4B, is the primary controller of the whole ADS. It replaced the original myRio-1900 controller because myRio-1900 does not support ROS dependencies [58]. The power is safely supplied to the Raspberry Pi using an X728 V2.3 UPS power circuit with two 18,650 lithium-ion batteries [59]. An ultra-thin cooling fan is also used on the Raspberry Pi to maintain its temperature within the normal range. The ROS node for real-time parameter tuning is implemented, written in Python, and installed on Raspberry Pi. With the aid of the ROS system, the control node will run the proposed algorithm for real-time parameter tuning of the PID gains. It receives the current steering value from the Arduino and generates the updated real-time PID gains for the steering controller.

The secondary controller, the Arduino, receives the desired steering angle and the PID parameter values from the Raspberry Pi controller. The Arduino Sends a PWM signal to the front motor driver to make the power steering motor reach the desired angle. The power steering motor receives the PWM signal from the motor driver and starts rotating the wheels. While rotating, the front angular potentiometer, connected to the power steering motor, changes its value and returns it to the Arduino. The Arduino receives the front encoder signal and checks whether the desired steering angle is reached. This process is controlled via a proposed PID controller to ensure the steering angle is accurately reached.

The proposed structure allows the modularity of adding new software and hardware layers. The rear motor and encoder can be connected to the Arduino hardware layer. Advanced sensors such as LiDAR and cameras can be connected to the Raspberry Pi. Mapping and localization algorithms can be easily implemented as ROS nodes on the Raspberry Pi. Therefore, the proposed layout is beneficial in the autonomous driving system.

### 6.2. Steering Angle Mapping and Experiment Setup

The closed loop of the steering control system relies heavily on the relationship between the steering angle of the motor’s shaft and the corresponding analog reading of the encoder. The relationship between the Arduino’s digital reading and the sensor’s analog reading can be obtained using the resolution of the ADC. However, the relationship between the analog voltage reading from the sensor and the motor’s rotation angle is unknown, and the relationship between the motor’s angle and the steering angle of the left and the right wheels is also unknown. Therefore, mapping experiments have been intensely conducted and analyzed to obtain these relationships. Moreover, the operating range of the seeing angle and the maximum and minimum steering angles are also undefined. Therefore, these angles should be practically measured and mapped to find these relationships.

In total, five parameters need to be measured in this experiment: the angular potentiometer voltage, the digital reading in the Arduino, the motor rotation angle, the left wheel steering angle, and the right wheel steering angle. This section discusses the setup and the tools used to measure all these five parameters accurately.

Regarding the voltage reading of the angular potentiometer, a multimeter is connected to the sensor’s output to measure the signal’s voltage. At the same time, the output of the potentiometer is connected to an Arduino analog input A0 to read the digital value of the sensor. The Arduino has a 10-bit analog-to-digital converter, so the output digital readings range from 0 to 1023 [60].

The most challenging part is measuring the three angles: the motor rotation angle, the left wheel steering angle, and the right wheel steering angle. This experiment aims to capture accurate photos of the angles and measure them by analyzing the digital image. It is performed as a set of steps to build a robust measurement system.

In the first step, three fixed zip ties were used for each angle to reference each angle. The zip tie for each angle is attached so that it points in the forward direction of the car. The zip ties are connected to three fixed rods on the scooter chassis; each rod is in line with the axis of rotation of each angle. A spirit-level tool has been used to ensure the fixed zip tie alignment, such that the fixed zip tie is perfectly parallel to the car’s chassis and points in the forward direction [61].

In the second step, a movable zip tie is attached to a movable rod in the chassis such that it rotates with the motor. Three rods have been chosen: a rod lies on the axis of rotation of the motor, a rod on the axis of rotation of the left wheel, and a rod on the axis of rotation of the right wheel. The movable zip tie is attached in a poison so that it intersects with the fixed zip tie at the axis of rotation of each angle. A cross-laser lever has been used to align the intersection point between the fixed and movable zip ties with the axis of rotation.

The cross-laser level projects two perpendicular intersecting laser lines, making a 90° angle. The fixed zip tie is aligned to one of the laser lines [62]. Then, the laser tool’s position is adjusted such that the laser line’s intersection point is in line with the axis of rotation of the motor. Finally, the movable zip tie is attached to align the second intersecting laser line. In this way, the intersection point between the fixed and the movable ties is aligned with the axis of rotation. The same steps are repeated for the left and right-wheel steering angles.

The third step is to fix three identical mobile phones at the bottom view for each angle. The cross-laser level tool has been used to align the camera lens of each phone with each angle. The phones are mounted on the scooter’s test bench using 3M double-sided tape to avoid any movement.

The fourth step is to enable remote camera access for the three phones from a fourth phone. This remote access has been achieved by installing AirDriod personal mobile client on all three phones: A, B, and C. We log in to the same AirDriod account on all three phones [63]. A fourth phone (Phone D) has been used as the controller of the three other phones (A, b, and C). The AirMirror app is installed on phone D, and we signed in to the same AirDroid account used on the three phones. After launching the AirMirror on phone D, we can see phones A, B, and C, and the camera for each phone can be triggered from phone D [64]. This way, the three angles can be remotely captured to measure the angles from the generated images.

### 6.3. Mapping Experiment Results and Data Collection

The experiment was conducted by changing the steering angle from the maximum right (5 V) to the maximum left (0 V). Small steps (less than 0.2 V) have been performed to ensure multiple readings. The photo for each angle has been captured and stored in the memory. Moreover, the multimeter reading and the digital value in the Arduino have also been stored. An angle meter application has been used to measure the angle between the fixed and the movable zip ties for the three angles [65].

**Figure 6:**
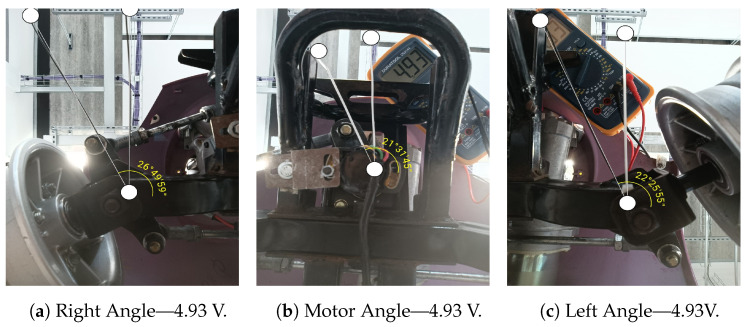
Steering angle mapping readings.

Figure 6 shows the photos captured for the three angles: the left steering angle for the left wheel, the motor rotation angle, and the right steering angle for the right wheel at specific encoder reading. The Supplementary Materials File presents more detailed captured photos (Figure S5).

The measured angles are written in the DMS (degrees, minutes, seconds) format. The DMS notation is used in applications that require a high level of precision. The path planning application requires high accuracy, so this notation has been used to get the most accurate angle readings. Equation (8) is used to convert the angles from the DMS format to decimal format [66].

(8) $$\mathrm{Decimal}\;\mathrm{Degrees}=\mathrm{Degrees}+\frac{\mathrm{Minutes}}{60}+\frac{\mathrm{Seconds}}{3600}$$

Table 5 shows all the measurements of the experiments. The first column represents the angular potentiometer readings obtained from the multimeter. It starts from 4.93 V to 1.01 V. The first finding in the readings is that the potentiometer range is from 4.93 V (at the maximum right) to 1.01 V (at the maximum left). The encoder readings from 0 V to 1.01 V always give the same steering angle because the chassis design hinders the wheels from going further than 1.01 V. The center position of the car is approximately at the encoder reading 3.13 V.

The second column represents the digital readings obtained from the Arduino. The Arduino has a 10-bit ADC, so the values should vary from 0 to 1023. However, the mechanism limitations make the values range from 210 (at the maximum left) to 1017 (at the maximum right), corresponding to the voltage between 1.01 V and 4.93 V.

The third column represents the motor rotation angle readings, the fourth column represents the left steering angle for the left wheel, and the fifth column represents the right steering angle for the right wheel, all in DMS format. Some values are positive, and some are negative. The positive sign is assumed for the angles of the left side turn, while the negative sign is assumed for all the angles in the right side turns, where the reference axis is in the middle, pointing in the forward direction.

Columns from six to eight represent the three angles but in decimal format. Equation (8) is used to map between the DMS values in columns three to five to decimal values written in columns from six to eight. The motor rotation angle range is from −21.529° at the right to 23.582° at the left. The left steering angle range is from −22.432° at the right to 31.430° at the left. The right steering angle range is from −26.833° on the right to 30.690° on the left.

### 6.4. Analysis of the Results of the Steering Angle Mapping

This section aims to find the relationship between the variables to be used in the kinematics layer in the proposed ROS system of the ADS. The required relationships are as follows:

- The motor rotation angle as a function of the sensor digital reading.
- The left steering angle as a function of the motor rotation angle.
- The right steering angle as a function of the motor rotation angle.

The machine learning algorithm to find the relationship between two or more continuous variables is called regression. The training data used to find the relationship between the variables are in Table 5. The Ordinary Least Square method (OLS) has been applied to find the parameters of the training model. This method can be used for linear, polynomial, and multiple regression problems with independent variables. The OLS method aims to minimize the sum of the square differences between the observed dependent variable, as in Table 5, and the predicted values by the polynomial [67].

Three important performance metrics show the quality of the chosen model: the mean square error (MSE), the R-squared metric, and the Prob(F-statistic). The MSE metric represents the means square error; the less the error, the better the model. The tolerance of the acceptable MSE is assumed to be less than or equal to 0.3. The R-squared measures the proportion variance in the dependent variable that the independent variables in the model can explain. A high R-squared indicates more variance and a best-fit description of the relationship. The probability of the F-statistic metric is a direct measure of the significance level of the model or the right fit for the training data. If the Prob(F-statistic) is less than 0.1, this indicates a significant solid relationship of the model [68].

#### 6.4.1. Motor Rotation Angle vs. Sensor Digital Reading

This section studies the relationship between the motor rotation angle as a dependent variable and the sensor digital reading as the independent variable. This relationship is needed in the feedback loop in the control system when the ADS system on the ROS sends the desired steering angle for the desired location of the scooter. The Arduino sends a signal to the front motor driver to enable the rotation of the steering motor. At this moment, the Arduino checks the digital reading from the steering motor encoder to check whether the desired steering angle of the motor is reached or not. Therefore, a relationship between the motor’s rotation angle and the encoder’s digital reading must be established.

The OLS algorithm has been applied to the training data of motor rotation angle in column six in Table 5 as a dependent variable, and the Arduino digital reading in column two in Table 5 as an independent variable. Multiple polynomials with different degrees have been tested from degree 2 to degree 10. Table 6 shows the algorithm’s results. The first column shows the degree of the polynomial; the second column is the number of the model parameters; the third column represents the mean square error (MSE); the fourth column shows the R-squared metric; and the last column is the Prob(F-statistic) metric.

The fourth-degree polynomial is the most suitable choice among all the models. The MSE of the fourth degree is significantly lower (0.2505) than that of all the other models, achieving a tolerance threshold of less than 0.3. The MSE has the minimum value at the fourth degree and starts increasing again when the polynomial degree increases, showing an over-fitting occurred for the high-degree polynomial, which is an undesirable response.

The R-squared of the fourth degree has the highest value (0.9988) and shows a very high variance in the relationship. The Prob(F-statistics) of the fourth degree has a very low value (less than 0.1) and shows a high significance level. The final relationship between the Arduino digital reading for the potentiometer sensor $D_{\mathrm{enc}}$ and the motor rotation angle $\theta_{m}$ is expressed in Equation (9) as a fourth-degree polynomial. The plot of the fourth-degree polynomial and the relationship between the degree of the polynomial and the MSE are presented in the Supplementary Materials File (Figure S6).

(9) $$\theta_{m}=0.0+0.0459D_{\mathrm{enc}}-0.0003D_{\mathrm{enc}}^{2}+0.0001D_{\mathrm{enc}}^{3}-7.41\times 10^{-8}D_{\mathrm{enc}}^{4}$$

#### 6.4.2. Left Steering Angle vs. Motor Rotation Angle

This section studies the relationship between the motor rotation angle as an independent variable and the left steering angle as the dependent variable. This relationship can help calculate the radius of turn of the left angle based on the steering motor angle, allowing the car position to be obtained after performing the steering.

The OLS algorithm was applied to the training data of motor rotation angle in column six in Table 5 as an independent variable, and the left steering angle in column seven in Table 5 as a dependent variable. Multiple polynomial degrees were tested from degree 1 to degree 10. Table 7 shows the algorithm’s results.

The seventh-degree polynomial is the most suitable choice among all the models. It offers a significantly lower MSE value (0.1315) than the lower degrees, achieving a tolerance threshold of less than 0.3. Despite the high degree of polynomial, it offers less MSE value, the error difference is insignificant, and the seventh degree provides less model complexity.

The R-squared of the seventh degree has the highest value (0.9996), indicating a good fit between the motor angle and the left steering angle. The Prob(F-statistics) of the 7th degree has a very low value (less than 0.1) and shows a high significance level. The final relationship between the left steering angle of the left wheel $\theta_{L}$ and the motor rotation angle $\theta_{m}$ is expressed in Equation (10) as a seventh-degree polynomial. The plot of the seventh-degree polynomial and the relationship between the degree of the polynomial and the MSE are presented in the Supplementary Materials File (Figure S7).

(10) $$\begin{array}{cc} \theta_{L}= & -0.2734+0.9598\theta_{m}-0.0123\theta_{m}^{2}+0.0041\theta_{m}^{3}\\ \; & +8.53\times 10^{-5}\theta_{m}^{4}-1.63\times 10^{-5}\theta_{m}^{5}-1.04\times 10^{-7}\theta_{m}^{6}+1.79\times 10^{-8}\theta_{m}^{7} \end{array}$$

#### 6.4.3. Right Steering Angle vs. Motor Rotation Angle

Similarly, this section obtains the relationship between the motor rotation angle as an independent variable and the right steering as the dependent variable. The OLS algorithm has been applied to the training data of motor rotation angle in column six in Table 5 as an independent variable, and the right steering angle in column eight in Table 5 as a dependent variable. Multiple polynomial degrees have been tested from degree 1 to degree 10. Table 8 shows the algorithm’s results.

The eighth-degree polynomial is the most suitable choice among all the models. It offers a significantly lower MSE value (0.2286) than the lower degrees, achieving a tolerance threshold of less than 0.3. Although the higher-degree polynomials offer better MSE values, the error difference is insignificant, with the possibility of overfitting. Therefore, the eighth degree provides less model complexity and archives the trade-off between the error and the complexity.

The R-squared of the eighth degree has a very high value (0.9993), indicating a good fit between the motor angle and the right steering angle. The Prob(F-statistics) of the eighth degree has a very low value (less than 0.1) and shows a high significance level. The final relationship between the right steering angle of the right wheel $\theta_{R}$ and the motor rotation angle $\theta_{m}$ is expressed in Equation (11) as an eighth-degree polynomial. The plot of the eighth-degree polynomial and the relationship between the degree of the polynomial and the MSE are presented in the Supplementary Materials File (Figure S8).

(11) $$\begin{array}{cc} \theta_{R}= & -1.2040+1.0667\theta_{m}-0.0190\theta_{m}^{2}+0.0011\theta_{m}^{3}+2.7361\times 10^{-4}\theta_{m}^{4}\\ \; & -3.7336\times 10^{-6}\theta_{m}^{5}-8.7724\times 10^{-7}\theta_{m}^{6}+4.6171\times 10^{-9}\theta_{m}^{7}+8.2089\times 10^{-10}\theta_{m}^{8} \end{array}$$

## 7. Steering Control Application Using the Proposed DHHO Algorithm

This section discusses the application of the PID controller to steering control using the proposed DHHO algorithm. The transient response of the PID-DHHO controller for the steering control system is evaluated by comparing its performance to the response from traditional methods, such as manual tuning, the ZN technique, and the original HHO algorithm.

### 7.1. The Configuration of the Steering Control System

**Figure 7:**
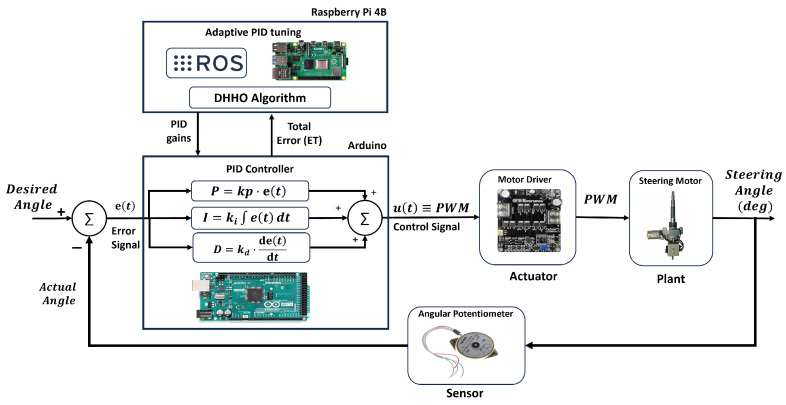
Steering control block diagram as a closed-loop system.

The steering control system is implemented as a closed-loop control system that minimizes error using a PID controller based on three actions: proportion, integral, and derivative. Figure 7 shows the block diagram of the closed-loop steering control system, which consists of four main blocks: the plant, actuator, sensor, and controller.

The plant of the steering control system is the power steering motor, which is a DC motor responsible for controlling the steering. The output of the steering motor is the steering angle, representing the controlled variable. The input signal of the steering motor is the motor input voltage as a PWM signal, representing the manipulated variable of the control system.

The sensor in the steering control system is the angular potentiometer, which is attached to the steering motor shaft to measure the rotation angle. The signal of the potentiometer represents the actual angle, where it is compared to the desired angle to generate the error signal, e(t) as in Equation (12). The error signal represents the difference between the desired angle and the actual steering angle measured by the sensor.

(12) e(t)=DesiredSteeringAngle−ActualSteeringAngle

The PID controller is implemented on the Arduino, receives the error signal, and generates the control signal. The control signal is equivalent to the manipulated signal (PWM signal), and it is generated based on three components: the proportional part, the integral part, and the derivative part. The proportional part achieves the concept of the proportional response to the error; if the error is high, the duty cycle will be high, and the motor moves faster; and if the error is small, the duty cycle of the PWM signal will be small, and the motor moves slower [69].

The integral part provides a final push to the control signal to eliminate the steady-state error. The proportional part cannot eliminate the steady-state error because the latter is too small to generate a control signal that can eliminate the final error. The derivative part predicts future errors and takes precautionary action to make the response faster toward the desired value. In other words, it improves the transient response. Equation (13) shows the relationship between all the PID components, the error signal, and the control signal [69]. This equation is implemented in C language on the Arduino controller.

(13) $$u\left(t\right)=K_{p}\cdot e\left(t\right)+K_{i}\cdot \int_{0}^{t}e\left(\tau \right)d\tau +K_{d}\cdot \frac{de(t)}{dt}$$

The actuator in this steering control system is the CYTRON MD30 motor driver, which receives the PWM control signal from the PID controller on the Arduino and generates the actuating signal that can control the steering motor’s speed and direction. The direction of the motor is controlled by a straightforward digital signal with two states: logic-high to rotate in one direction and logic-low to rotate in the other direction. The speed of the motor is controlled using the PWM signal, which is obtained from the PID controller. The motor’s speed and direction can be easily controlled via the PWM signal, but the question is how to control the steering angle.

The short answer to this question is the PWM combined with a feedback element. Although the PWM is mainly used to control the speed, the steering control indirectly relies on the PWM signal. The concept of controlling the angle is to set the PWM signal to a specific duty cycle to make the motor rotate in the direction of the desired angle with a particular speed. The closer the motor moves towards the desired angle, the less the duty cycle of the PWM signal. In other words, if the desired angle is further, the motor moves faster at the beginning, and if the motor is close to the desired angle, the motor moves slower towards the desired angle.

The gains of the PID controllers are dynamically tuned using the proposed DHHO algorithm, which is implemented as an ROS node on the Raspberry Pi controller. The PID tuning of the scooter’s steering control is formulated as an optimization problem. Any optimization problem should define two main aspects: the solution format and the objective (fitness) function. The solution to the PID tuning problem is the three PID gains of the PID controller: $K_{p},K_{i}$, and $K_{d}$, representing a three-dimensional optimization problem.

The objective function of the problem can be represented as a weighted sum of the squared error and the maximum overshoot as in Equation (14). The squared error term represents the sum of the square of the error between the desired steering angle and the actual steering angle reading from the encoder over a specific time T. The square error is used instead of the sum of the errors to avoid negative-value errors and cancel positive errors during the summation process. The second term represents the maximum overshoot, the maximum difference between the desired value and the actual steering angle measured by the encoder over time T. It is required to keep the error and the maximum overshoot at the minimum value to obtain the best performance. The weights $w_{1}$ and $w_{2}$ are the weights for squared error and maximum overshoot metrics, set to 0.7 and 0.3, respectively.

(14) $$\mathrm{ET}=w_{1}\times \mathrm{SE}+w_{2}\times \mathrm{MO}$$

where

$$\begin{array}{cc} \mathrm{ET} & =\mathrm{the}\;\mathrm{total}\;\mathrm{error}\\ \mathrm{SE} & =\sum_{t=0}^{T}{\left(e\left(t\right)\right)}^{2},\\ \mathrm{MO} & =\max_{t\leq T}\left(\frac{e(t)}{\mathrm{desired}\;\mathrm{Angle}}\right) \end{array}$$

The main objective is to apply the proposed DHHO algorithm to minimize the total error of the objective function. The algorithm is applied to the steering control in real time, reading the final error from the Arduino and re-tuning the parameters until it reduces the error to the minimum value and improves the performance metrics.

### 7.2. Adaptive PID Tuning Using DHHO Algorithm

This section explains how the DHHO algorithm is applied to generate PID parameters for the steering control the system.

#### 7.2.1. Experiment Setup

**Figure 8:**
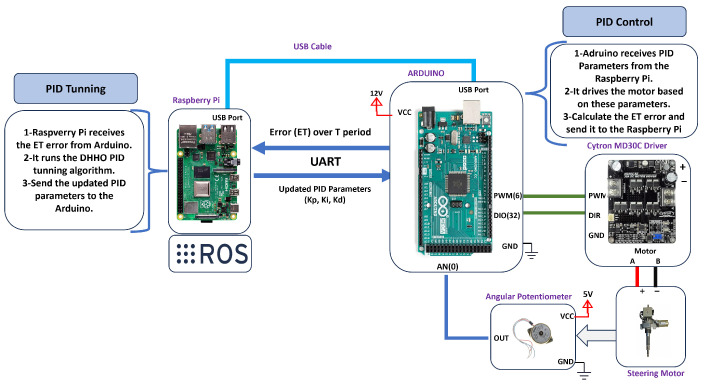
The hardware setup of the PID tuning of the steering control.

Figure 8 displays the setup of the experiment and how the PID parameters are tuned in real time using the proposed DHHO algorithm. The setup is based on the communication between the Arduino and the Raspberry Pi. The Arduino is connected to the motor driver to send the control signal to the steering motor. A potentiometer is connected to the Arduino to read the current steering angle and send it to the Arduino through an analog signal. The Arduino is connected to the Raspberry Pi via a USB cable over the UART serial communication protocol.

The sequence of the execution is as follows: the desired angle is set inside the Arduino. Then, the error between the desired value and the encoder reading is calculated. With initial random PID gains, the PID controller equation is performed on the Arduino and generates the control signal based on the error. The control signal is in the form of a PWM signal that is sent to the steering motor via the motor driver. The encoder transmits the current angle reading, and the loop contains until the steering angle settles within a specific value after a period T.

The squared errors between the desired angle and the encoder readings are summed over the period T to obtain the SSE value. The maximum difference between the encoder reading and the desired steering angle is used to calculate the maximum overshoot. The final error is calculated based on Equation (14), and the total error (ET) value is sent from the Arduino to the Raspberry Pi.

On the Raspberry Pi, the proposed DHHO algorithm is implemented as a ROS node that subscribes to Arduino and receives the final ET error. This value represents the fitness of the current PID controller parameters. Then, the algorithm runs another cycle to calculate the updated PID parameters based on the received error. The Raspberry Pi publishes and sends the updated PID parameters to the Arduino. The cycle is repeated until the total error reaches the minimum value the proposed DHHO can reach.

#### 7.2.2. PID Parameter Tuning Results

The PID parameters obtained by the DHHO algorithm are 14.1019, 86.4520, and 1.0117 for $K_{p},K_{i}$, and $K_{d}$, respectively. The performance of the proposed PID-DHHO controller is evaluated and compared with three other controllers tuned using the manual tuning technique, the Ziegler–Nichols (ZN) method, and the traditional HHO algorithm.

The obtained PID parameters using the traditional HHO algorithm are 33.1505, 67.012, and 0.9064 for $K_{p},K_{i}$, and $K_{d}$, respectively. The PID gains obtained using the manual tuning method are $K_{p}=5,K_{i}=30$, and $K_{d}=0.15$ [70]. Furthermore, the PID gains of the ZN method are 18, 31.92, and 2.45 for $K_{p},K_{i}$, and $K_{d}$, respectively, [71]. The PID gains for all the methods are displayed in Table 9.

### 7.3. Transient Response Analysis for All the PID Controllers

After tuning, the system’s performance is tested using four PID controllers: PID1 (manually tuned controller), PID2 (ZN method), PID3 (HHO algorithm), and PID4 (proposed DHHO algorithm). In total, five performance metrics were used to compare the quality of the transient response of the steering control process: the rise time (tr), the peak time (tp), the settling time (ts), the maximum overshoot (Mp), and the steady state error (ess). These metrics are essential to judge whether the system’s response is accurate.

#### 7.3.1. Performance Metrics

The rise time (tr) is the time required first to reach 90% of the desired value. This metric is significant as it measures how quickly the system generates the initial response to a change in steering input. Peak time (tp) is the time taken to reach the first peak of the steering angle. It is an indicator of the speed of the initial system response but is also associated with the oscillations. The settling time (ts) is the time required for the steering system to settle within a certain percentage of (2% or 5%) of the final steering angle. It indicates how quickly the system settles and stabilizes after input change [72].

The maximum overshoot (Mp) shows how much the response exceeded the desired steering angle, which is crucial for steering control as excessive overshoot can lead to instability and inaccurate steering angles [73]. This metric is critical because it reflects the system’s stability and ability to avoid over-correcting. A high overshoot can cause the steering angle to exceed the desired value, potentially leading to significant deviations from the planned path. This high value can be particularly problematic if the error accumulates over time, preventing the system from returning quickly to the correct steering angle. Minimizing overshoot is essential to avoid undesirable responses and ensure the vehicle remains on its intended path.

The steady-state error (ess) is the difference between the desired steering angle and the actual final one, which is essential for assessing the system’s accuracy in reaching the desired steering angle. A low steady-state error ensures that the steering angle closely matches the desired angle, which is crucial for following a planned path accurately and reaching the destination [74]. High accuracy is essential to avoid deviations from the intended path, which can lead to safety issues or failure to reach the destination.

The metrics ts, tr, and tp indicate the system’s speed, while the ess and Mp indicate the system’s accuracy. The control process represents a trade-off between accuracy and speed. In steering control and path planning for autonomous driving systems, reaching the destination safely and accurately is more important than quickly. Therefore, Mp and ess have more priority in the control process.

#### 7.3.2. Experiment Setup and Data Collection

The transient response for each controller is measured and monitored for different steering angles, starting from −21° to 21 degrees. First, the steering angles are set to zero (center) at the beginning of each experiment at each steering angle trial. Second, the desired angle is set internally at the Arduino code for each controller at a specific steering angle. Third, the system starts, and the control system runs, in which the desired steering angle is compared to the current angle reading from the steering encoder sensor, generating the error signal. The PID controller generates the control signal to minimize this error to zero.

**Figure 9:**
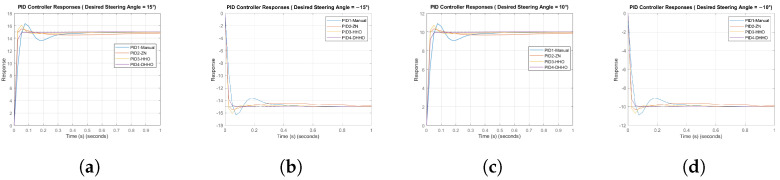
Transient response of different setpoints using PID controller. (**a**) Steering angle 15°. (**b**) Steering Angle −15°. (**c**) Steering angle 10°. (**d**) Steering Angle −10°.

During the running, the steering angle is collected at different time steps starting from zero until the system terminates; we stop the system after 5 s from applying the desired angle, which is sufficient for all the systems to stabilize. The steering angle values and the corresponding time stamps are saved to be plotted and analyzed. The collected data has been stored and plotted using Matlab R2024a software as seen in Figure 9. The detailed collected readings for each controller’s steering angle response at 20 timestamps for different steering angles are presented in the Supplementary Materials File (Table S1).

#### 7.3.3. Results Analysis and Discussion

The collected results were analyzed using Matlab, and the performance metrics are calculated for each steering angle for each controller. The results show that all the PID controllers generate the same response regardless of the setpoint, showing the consistency and the reliability of the tuning obtained by all the algorithms, as seen in Figure 9. Table 10 shows the performance metrics for each controller.

**Figure 10:**
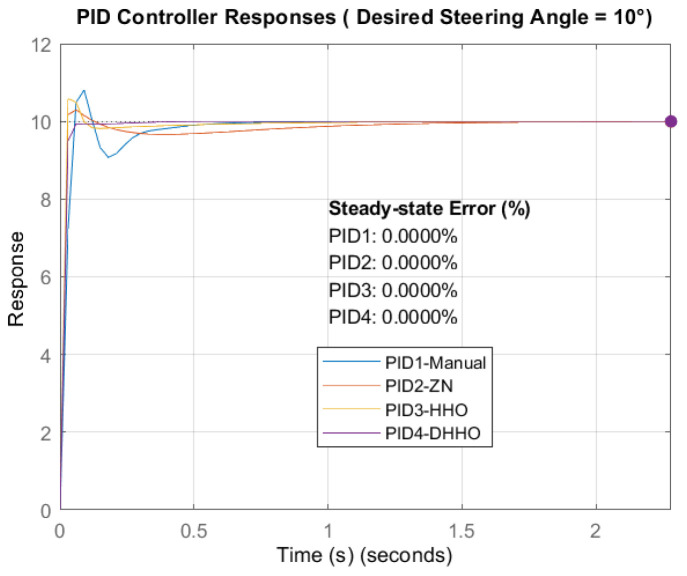
Transient response of the steering control using PID controller (Steady-state error for steering control).

Regarding the steady-state error (ess), all the controllers managed to eliminate the steady-state error after five seconds from applying the desired angle, showing a zero-ess, as presented in Figure 10. The zero error behavior is due to the integral component of the PID controller, which is mutual in the four controllers used in the experiment. This merit is one of the main reasons why PID controllers, especially the Integral component, are viral in the control systems.

**Figure 11:**
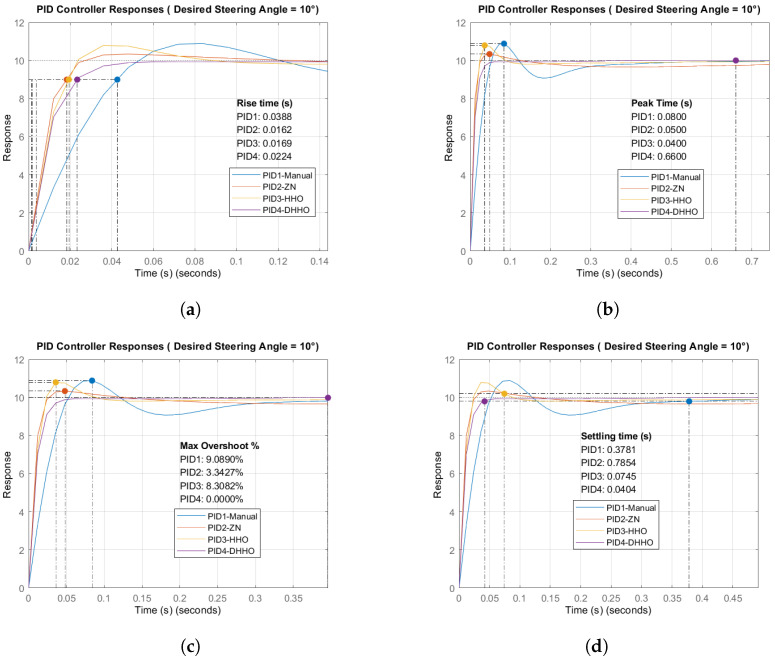
Transient response of the steering control using PID controller. (**a**) Rise time metric for steering control. (**b**) Peak time metric for steering control. (**c**) Maximum Overshoot for steering control. (**d**) Settling time for steering control.

Figure 11a shows a close-up look at the rise time for all the PID controllers. The ZN and the HHO algorithms consistently provide the lower rise time, approximately 0.017 s, indicating faster response than the Manual and the proposed DHHO algorithms. However, the proposed DHHO algorithm also shows a competitive rise time of 0.022 s, which is too close to the ZN and HHO algorithms.

Figure 11b shows all PID algorithms’ peak time calculations. The HHO algorithm has the lowest peak time, 0.04 s, compared to the other algorithms. The proposed DHHO algorithm proved a peak time of 0.66 s, which seems too slow compared to the other three algorithms. However, this peak time is reached at the end of the response after the settling time because the DHHO algorithms’ response has no oscillations. Therefore, slow peak time is not a disadvantage in this case.

The proposed DHHO algorithms stand out with a significant settling time of 0.0404 s, indicating rapid stabilization, as seen in Figure 11d. This metric is a crucial advantage as the system reaches stability quickly and accurately. The HHO algorithm shows a competitive setting time of 0.075 s, which is considered an acceptable response. Manual tuning offers a settling time of 0.378, better than that obtained from the ZN method of 0.785 s.

The proposed DHHO algorithm dominated the performance and outperformed all the algorithms with a zero maximum overshoot, as indicated in Figure 11c. The system’s response is close to the overdamped response with no oscillations. This smooth response comes at the cost of a reduction in speed, as previously discussed. However, the fast stability with zero overshoot highlights the superiority of the proposed DHHO algorithm.

## 8. Overall Discussion

The main objective of this paper is to improve the steering performance of autonomous personal mobility scooters using a proposed algorithm named the differential Harris Hawks optimization (DHHO) algorithm. The findings of the theoretical and practical experiments conducted in this research can be formulated as follows:

### 8.1. The Theoretical Benchmark Testing of the DHHO Algorithm

The DHHO algorithm is proposed as an improved version of the original HHO algorithm. The objective of the DHHO algorithm is to improve the exploration and the global search of the HHO algorithm by deploying the DE/rand/1 mutation rule. The exploration of both algorithms has been validated on different dimensions, starting from 10D to 100D. The simulation results showed that the mean variance of the DHHO population is 6.0904303E+01, which is significantly greater than the variance of the HHO population (4.8669746E+01). The mean SD of the DHHO is 7.749476E+00, which is greater than the SD of HHO (6.952564E+00). Higher variance and SD in the population indicate better exploration capability of the DHHO algorithm and higher diversity compared with the original HHO algorithm.

The DHHO algorithm is validated on CEC2020/2021 benchmark functions and compared with the other algorithms used in the literature for steering control: the PSO, BAS, CMAES, and HHO algorithms. The DHHO algorithm ranked first on the Friedman test, and it showed a *p*-value less than 0.05 in all the paired comparisons with the PSO, BAS, CMAES, and HHO algorithms, indicating significant performance of the DHHO algorithm.

### 8.2. Mapping and Calibration of Steering Angle and the Encoder

The steering hardware of the personal mobility scooter is improved by introducing a potentiometer as a steering encoder mapped with the motor’s shaft steering angle. A practical experiment has been conducted to find the relationship between the motor steering angle and encoder reading. Regression-supervised learning is performed on the collected results from the mapping experiment, and a fourth-degree polynomial is obtained with an F-statistic of 3.59E-25 and R-squared of 0.9983.

These results of this model mean that 99.83% of the variance in the steering angle can be explained by the encoder reading in the deduced equation. In other words, the regression model fits the data very well, with only 0.17% of the steering angle variance unexplained by the equation, avoiding an overfitting situation. This relationship is significant because the f-statistic is less than 0.05 (3.59e-25), indicating a reliable equation that can be used in the steering control application in the feedback control loop.

### 8.3. Application of the DHHO Algorithm on Steering Control

The DHHO algorithm is applied for the steering control for dynamic PID tuning, and it is compared with the traditional manual tuning, ZN method, and original HHO algorithm. The results of the four controllers showed exciting behavior in the four steering control systems regarding the rise time (tr), peak time (tp), settling time (ts), maximum overshoot (Mp), and steady-state error (ess). All the controllers managed to eliminate the ess due to the integral part of the PID controller. The transient response of the DHHO controller shows a slower initial response in terms of the tr (0.0224 s) and tp (0.66 s) compared with the other three controllers, where the ZN controller has a tr of 0.0162 s and a tp of 0.05 s. On the other hand, the DHHO algorithm eliminates Mp compared to the other three controllers. Moreover, DHHO has the smallest ts of 0.0404 s, while the ts for the manual, ZN, and HHO controllers are 0.3781 s, 0.7854 s, and 0.0745 s, respectively.

The interpretation of this phenomenon is that the slower the tr and tp, the more time the DHHO controller has to make precise decisions to avoid oscillations and overshoot such that it can settle at the goal in a faster settling time. Therefore, this slow initial response of the DHHO indicates a cautious response, which enables it to avoid oscillations and reduces the Mp and ts, which is a desirable accurate response. On the other hand, the other three controllers have a more rapid initial response with a small rise time and peak time. This quick initial response led to oscillations and high overshoot. Therefore, these systems take a longer settling time to stabilize and reach the stable case to overcome these oscillations, which explains the high settling time and overshoot.

The overall performance of the proposed DHHO algorithm managed to eliminate the overshoot and steady-state error with a smooth response free of oscillations, which is highly advantageous in providing a stable response. Although the rise and peak times are not the best compared to the other algorithms, they are still competitive and acceptable, especially after considering the remarkable performance of the other metrics (Mp and ts). Furthermore, the ts of the proposed scooter in [24] was around 0.580 s, larger than the one obtained by the DHHO controller (0.0404 s), showing significant improvement of the proposed methodology.

## 9. Conclusions

In this paper, we achieve improved steering control of the Ackermann mobility scooter using a PID controller optimized by a new algorithm named the Differential Harris Hawks Optimization (DHHO) algorithm.

First, a practical experiment was conducted on the power steering mechanism of the scooter to obtain the relationship between the steering motor angle and the steering encoder. Supervised learning and regression techniques were applied to help determine this relationship, which is a crucial equation in steering control. Statistical analysis was performed on these relationships to find the significance level and the reliability of the equations, and all achieved a *p*-value of less than 0.05.

The DHHO algorithm is proposed to improve the diversity of the original Harris Hawks Optimization (HHO) algorithm by introducing a Hawks mutation operator in the exploration phase instead of the Harris Hawks perch technique. This modification improved the diversity of the population compared to the HHO, decreasing the probability of becoming trapped into a local minimum. The mean variance of the DHHO population increased by 25.14%, and the mean SD increased by 11.74% compared with the HHO algorithm, indicating improved diversity in the DHHO population compared with the original HHO algorithm.

The DHHO algorithm is compared with the traditional HHO, PSO, BAS, and CMAES algorithms and validated on CEC2020/2021 benchmark functions. The proposed DHHO algorithm outperforms all the other algorithms, achieving first place in the Friedman test with significant *p*-values. In the 10-dimension case, the DHHO algorithm achieved better significant performance by 98.05%, 99.4141%, 99.8047%, and 99.8047% confidence compared with the PSO, CMAES, BAS, and HHO algorithms, respectively. Regarding the 20-dimension case, the DHHO algorithm achieved better significant performance by 99.0234%, 99.4141%, 99.8047%, and 91.6016% confidence compared with the PSO, CMAES, BAS, and HHO algorithms, respectively. These simulation results of the benchmark testing demonstrate that the DHHO algorithm outperformed the PSO, CMAES, BAS, and HHO algorithms.

The proposed DHHO algorithm is practically applied to the PID tuning of the steering control in the Ackermann scooter. The steering performance of the DHHO was evaluated compared with traditional PID, Ziegler–Nichols (ZN)-PID, and HHO-PID controllers. The statistics proved that the DHHO algorithm eliminates the maximum overshoot (0%) and the steady-state error (0%). Moreover, the DHHO gives the best settling time (ts); ts is improved by 89.32%, 94.85%, and 45.76% compared with the manual-PID, ZN-PID, and HHO-PID controllers. The steering settling time response is also enhanced by around 93% compared to the settling time of the original scooter performance.

In the future, the proposed DHHO algorithm can be extended to control the speed of the scooter’s rear motor by applying the DHHO algorithm to dynamically tune the gains of a PID controller that controls the speed (RPM) of the rear motor. The rear motor speed will replace the steering angle in the proposed control system, and the motor speed can be measured using a rotary inductive encoder attached to the rear wheels of the scooter. DHHO algorithm can also be applied to control the steering of different autonomous vehicles such as Unmanned Aerial Vehicles (UAVs), Autonomous Surface Vehicles (ASVs) such as ships, and autonomous underwater vehicles (AUVs).

The DHHO algorithm can also be applied to different optimization problems in other fields, such as truss design optimization, heat exchanger design, battery management systems, etc. Further improvements can be made to the parameters of the DHHO algorithm, such as employing adaptive techniques like that used in the JADE algorithm to dynamically change the mutation constant F and the crossover probability CR instead of static ones. The significant results of this research render the proposed DHHO algorithm a promising starting point for future research and a step toward improving automated driving.

## Figures and Tables

**Table 1:** Variance comparison between the HHO and DHHO exploration techniques.

Dim. Size	Method Name	Variance	SD
10 Dim	HHO	4.795357E+01	6.902057E+00
	DHHO	5.648867E+01	7.502079E+00
20 Dim	HHO	4.830405E+01	6.915374E+00
	DHHO	5.924026E+01	7.677845E+00
30 Dim	HHO	4.735670E+01	6.873195E+00
	DHHO	6.347248E+01	7.929779E+00
40 Dim	HHO	4.925848E+01	7.007395E+00
	DHHO	5.955556E+01	7.668104E+00
50 Dim	HHO	4.786387E+01	6.906827E+00
	DHHO	6.066903E+01	7.747510E+00
60 Dim	HHO	4.923419E+01	6.999774E+00
	DHHO	6.178129E+01	7.808221E+00
70 Dim	HHO	5.050641E+01	7.077303E+00
	DHHO	5.995979E+01	7.733081E+00
80 Dim	HHO	4.905733E+01	6.979320E+00
	DHHO	6.471879E+01	8.034884E+00
90 Dim	HHO	4.806122E+01	6.925808E+00
	DHHO	6.252086E+01	7.892397E+00
100 Dim	HHO	4.910164E+01	6.988329E+00
	DHHO	6.063630E+01	7.751442E+00
Mean	HHO	4.8669746E+01	6.9575382E+00
	DHHO	6.0904303E+01	7.7745342E+00

**Table 2:** Parameter Settings for the Algorithms.

Algorithm	Parameter	Value
All algorithms	population size (popSize)	30
PSO [35,51]	personal learning coefficient $c_{1}$	1
	global learning coefficient $c_{2}$	1
	inertia weight *w*	0.3
CMAES [52]	parents number μ	popSize/2
	change rate of the covariance matrix $c_{cov}$	$\frac{2}{{\left(nd+\sqrt{2}\right)}^{2}}$
	cumulation for distribution $C_{c}$	$\frac{4}{nd+4}$
	damping rate for the step size $d_{\sigma }$	$c_{\sigma }^{-1}+1$
		where nd is the dimSize
BAS [53]	step size initial value $\delta^{1}$	0.5
	step shift coefficient $\delta_{0}$	0.00
	step coefficient rate $\eta_{\delta }$	0.95
	antennae length rate coefficients $\eta_{d}$	0.95
	antennae length initial value $d^{1}$	2
	antennae length shift coefficient $d_{0}$	0.01
HHO [35]	escaping energy limit $E_{limit}$	2
	randomly varies [−2,2]	
DHHO	escaping energy limit $E_{limit}$	2
	randomly varies [−2,2]	
	crossover probability CR	0.5
	Mutation factor *F*	0.5

**Table 3:** The error results for the 30 runs: best, worst, mean, median, std for the 10-Dim and 20-Dim CEC2020/2021 benchmark functions for all the algorithms.

Fun.	Alg.	Error (10-Dim)					Error (20-Dim)				
No.	Name	Best	Worst	Median	Mean	SD	Best	Worst	Median	Mean	SD
F1	PSO	3.16E+05	3.68E+09	5.34E+08	6.36E+08	7.68E+08	1.77E+09	1.46E+10	6.31E+09	6.57E+09	2.84E+09
	CMAES	9.70E+03	6.81E+09	3.85E+09	3.95E+09	1.49E+09	5.08E+02	2.27E+10	1.05E+10	8.55E+09	7.79E+09
	BAS	1.15E+10	3.67E+10	2.30E+10	2.33E+10	6.64E+09	3.44E+10	9.12E+10	5.96E+10	6.20E+10	1.31E+10
	HHO	5.84E+06	1.78E+09	3.15E+08	4.30E+08	4.30E+08	2.56E+07	1.12E+09	1.42E+08	1.82E+08	1.93E+08
	DHHO	1.46E+07	1.50E+09	1.05E+08	1.97E+08	2.94E+08	7.22E+07	1.13E+09	2.27E+08	3.03E+08	2.20E+08
F2	PSO	3.52E+02	1.52E+03	9.16E+02	9.24E+02	2.94E+02	1.61E+03	3.76E+03	2.48E+03	2.48E+03	4.60E+02
	CMAES	1.19E+03	2.07E+03	1.69E+03	1.70E+03	2.02E+02	3.94E+03	5.12E+03	4.54E+03	4.61E+03	2.95E+02
	BAS	1.74E+03	2.63E+03	2.25E+03	2.25E+03	2.45E+02	4.93E+03	6.22E+03	5.68E+03	5.66E+03	4.12E+02
	HHO	6.04E+02	1.97E+03	1.24E+03	1.21E+03	3.86E+02	1.83E+03	4.46E+03	2.81E+03	2.83E+03	5.88E+02
	DHHO	4.79E+02	1.55E+03	9.68E+02	9.46E+02	2.76E+02	1.56E+03	3.37E+03	2.57E+03	2.54E+03	4.14E+02
F3	PSO	1.91E+01	8.21E+01	4.62E+01	4.73E+01	1.45E+01	8.56E+01	2.96E+02	1.69E+02	1.77E+02	4.57E+01
	CMAES	1.15E+01	2.01E+01	1.41E+01	1.41E+01	1.81E+00	2.76E+01	9.35E+01	3.20E+01	3.47E+01	1.16E+01
	BAS	2.36E+02	6.18E+02	4.31E+02	4.40E+02	9.69E+01	8.60E+02	1.84E+03	1.43E+03	1.42E+03	2.16E+02
	HHO	6.14E+01	1.45E+02	1.11E+02	1.06E+02	2.16E+01	1.93E+02	3.61E+02	2.87E+02	2.88E+02	3.66E+01
	DHHO	4.65E+01	1.46E+02	8.96E+01	8.72E+01	2.06E+01	1.53E+02	3.54E+02	2.74E+02	2.69E+02	5.46E+01
F4	PSO	9.05E+00	3.27E+04	1.05E+03	2.99E+03	6.27E+03	5.95E+02	5.38E+04	6.46E+03	1.05E+04	1.18E+04
	CMAES	1.64E+02	5.67E+03	1.68E+03	1.80E+03	1.35E+03	2.08E+03	1.87E+05	4.12E+04	5.81E+04	4.70E+04
	BAS	6.88E+03	1.60E+07	2.90E+06	4.21E+06	4.32E+06	2.10E+06	2.48E+07	1.23E+07	1.19E+07	5.57E+06
	HHO	4.89E+00	8.81E+02	2.24E+01	8.18E+01	1.99E+02	2.69E+01	2.11E+03	5.03E+01	1.38E+02	3.79E+02
	DHHO	3.59E+00	8.90E+01	1.20E+01	1.74E+01	1.66E+01	2.01E+01	3.66E+02	5.90E+01	9.93E+01	9.60E+01
F5	PSO	3.36E+03	1.82E+06	4.11E+05	5.17E+05	4.77E+05	1.51E+05	9.35E+06	1.77E+06	2.74E+06	2.22E+06
	CMAES	1.40E+04	1.27E+06	2.12E+05	2.76E+05	2.95E+05	5.34E+05	3.21E+07	8.63E+06	1.05E+07	7.16E+06
	BAS	8.07E+03	6.19E+07	9.00E+06	1.37E+07	1.42E+07	2.36E+07	4.41E+08	1.08E+08	1.45E+08	1.07E+08
	HHO	8.49E+03	6.36E+05	2.62E+05	2.73E+05	2.38E+05	1.75E+05	4.85E+06	1.60E+06	1.77E+06	1.09E+06
	DHHO	1.76E+03	3.86E+05	3.13E+04	8.06E+04	1.07E+05	4.90E+04	2.70E+06	1.09E+06	1.05E+06	7.78E+05
F6	PSO	1.22E+02	7.84E+02	2.98E+02	3.48E+02	1.74E+02	4.57E+02	1.39E+03	8.22E+02	8.80E+02	2.86E+02
	CMAES	3.12E+02	7.18E+02	4.14E+02	4.51E+02	1.15E+02	9.00E+02	1.75E+03	1.24E+03	1.24E+03	2.39E+02
	BAS	6.11E+02	2.46E+03	1.13E+03	1.20E+03	4.41E+02	1.72E+03	4.75E+03	2.92E+03	2.97E+03	7.03E+02
	HHO	1.10E+02	5.68E+02	2.81E+02	2.97E+02	1.35E+02	5.63E+02	1.75E+03	9.64E+02	1.00E+03	2.83E+02
	DHHO	2.92E+01	3.56E+02	1.59E+02	1.67E+02	8.54E+01	1.68E+02	1.27E+03	4.67E+02	5.04E+02	2.43E+02
F7	PSO	2.37E+02	1.89E+06	1.42E+04	1.15E+05	3.52E+05	3.03E+04	1.20E+07	8.30E+05	1.93E+06	2.81E+06
	CMAES	3.38E+03	3.54E+06	2.93E+05	7.07E+05	1.01E+06	4.29E+05	3.23E+07	3.78E+06	6.63E+06	7.98E+06
	BAS	7.11E+03	3.07E+07	1.83E+06	4.39E+06	7.13E+06	9.52E+06	5.34E+08	6.99E+07	9.95E+07	1.08E+08
	HHO	2.83E+03	2.65E+06	8.73E+04	3.40E+05	6.36E+05	6.80E+04	2.24E+06	5.16E+05	5.78E+05	4.71E+05
	DHHO	1.03E+03	3.16E+04	8.91E+03	1.11E+04	8.88E+03	9.90E+03	1.04E+06	2.80E+05	3.42E+05	2.60E+05
F8	PSO	5.72E+01	1.71E+03	1.34E+02	2.71E+02	4.15E+02	3.31E+02	4.16E+03	1.76E+03	2.04E+03	1.40E+03
	CMAES	1.00E+02	1.92E+03	1.00E+02	4.10E+02	5.90E+02	1.00E+02	5.08E+03	4.71E+03	4.44E+03	1.04E+03
	BAS	1.04E+03	2.90E+03	2.06E+03	2.03E+03	5.54E+02	3.65E+03	6.94E+03	5.99E+03	5.87E+03	7.97E+02
	HHO	9.34E+01	1.69E+02	1.29E+02	1.33E+02	1.76E+01	1.23E+02	4.08E+03	1.93E+02	1.33E+03	1.59E+03
	DHHO	1.11E+02	1.64E+02	1.21E+02	1.23E+02	1.06E+01	1.32E+02	3.35E+03	1.53E+02	3.77E+02	7.97E+02
F9	PSO	1.09E+02	5.40E+02	4.62E+02	4.19E+02	1.17E+02	7.14E+02	1.27E+03	9.04E+02	9.28E+02	1.24E+02
	CMAES	3.79E+02	4.24E+02	4.13E+02	4.11E+02	1.01E+01	5.71E+02	6.54E+02	6.11E+02	6.09E+02	1.96E+01
	BAS	5.06E+02	8.59E+02	6.14E+02	6.38E+02	1.03E+02	1.00E+03	1.75E+03	1.36E+03	1.39E+03	2.11E+02
	HHO	1.13E+02	5.07E+02	4.01E+02	3.78E+02	1.02E+02	5.58E+02	1.03E+03	7.09E+02	7.24E+02	1.21E+02
	DHHO	1.47E+02	4.01E+02	3.75E+02	3.61E+02	5.37E+01	4.88E+02	6.26E+02	5.38E+02	5.39E+02	3.12E+01
F10	PSO	3.98E+02	6.17E+02	4.55E+02	4.64E+02	5.18E+01	6.20E+02	1.20E+03	8.29E+02	8.75E+02	1.74E+02
	CMAES	3.98E+02	9.87E+02	6.06E+02	6.16E+02	1.36E+02	5.11E+02	2.17E+03	1.20E+03	1.29E+03	4.73E+02
	BAS	1.01E+03	4.25E+03	1.77E+03	2.12E+03	7.59E+02	4.86E+03	2.39E+04	1.07E+04	1.15E+04	4.31E+03
	HHO	4.02E+02	5.92E+02	4.57E+02	4.68E+02	4.04E+01	4.93E+02	6.53E+02	5.42E+02	5.49E+02	3.93E+01
	DHHO	4.11E+02	4.89E+02	4.55E+02	4.57E+02	1.45E+01	4.72E+02	6.05E+02	5.34E+02	5.34E+02	3.19E+01

**Table 4:** Statistical Analysis for all the algorithms for the 10-Dim and the 20-Dim benchmark functions. DHHO is used as a reference for all paired comparisons. In the Sign Test: ‘+’ represents the number of functions where the DHHO is better, and ‘=’ means draw. In the Wilcoxon: R+ > R− means the DHHO is better. In the Friedman test, a smaller mean rank means a better algorithm. The significance level α is between 0.05 and 0.1. The results are significant if *p*-value < α.

Dim	Metric	Alg.	Friedman Test				Sign Test	Wilcoxon Test			
			SumRanks	MeanRanks	Rank	*p*-Value	+/=/−	R+	R−	*p*-Value	H
10 Dim	Mean	PSO	28	2.8	3	6.61E-06	+8/=0/−2	50	5	0.019531	TRUE
		CMAES	34	3.4	4		+9/=0/−1	53	2	0.005859	TRUE
		BAS	50	5	5		+10/=0/−0	55	0	0.001953	TRUE
		HHO	25	2.5	2		+10/=0/−0	55	0	0.001953	TRUE
		DHHO	13	1.3	1		NA	NA	NA	NA	NA
	Median	PSO	28	2.8	3	2.18E-05	+8/=0/−2	48	7	0.037109	TRUE
		CMAES	31	3.1	4		+8/=0/−2	51	4	0.013672	TRUE
		BAS	50	5	5		+10/=0/−0	55	0	0.001953	TRUE
		HHO	27	2.7	2		+10/=0/−0	55	0	0.001953	TRUE
		DHHO	14	1.4	1		NA	NA	NA	NA	NA
20 Dim	Mean	PSO	27	2.7	3	7.67E-06	+8/=0/−2	52	3	0.009766	TRUE
		CMAES	35	3.5	4		+9/=0/−1	53	2	0.005859	TRUE
		BAS	50	5	5		+10/=0/−0	55	0	0.001953	TRUE
		HHO	24	2.4	2		+9/=0/−1	45	10	0.083984	TRUE
		DHHO	14	1.4	1		NA	NA	NA	NA	NA
	Median	PSO	27	2.7	3	1.07E-05	+8/=0/−2	52	3	0.009766	TRUE
		CMAES	35	3.5	4		+9/=0/−1	53	2	0.005859	TRUE
		BAS	50	5	5		+10/=0/−0	55	0	0.001953	TRUE
		HHO	23	2.3	2		+8/=0/−2	43	12	0.130859	FALSE
		DHHO	15	1.5	1		NA	NA	NA	NA	NA

**Table 5:** Measurements of the steering angle experiment.

V	D	$\theta_{m}$ (DMS)	$\theta_{L}$ (DMS)	$\theta_{R}$ (DMS)	$\theta_{m}$ (°)	$\theta_{L}$ (°)	$\theta_{R}$ (°)
4.93	1017	$-21^{\circ }31^{\prime }45^{\prime \prime }$	$-22^{\circ }25^{\prime }55^{\prime \prime }$	$-26^{\circ }49^{\prime }59^{\prime \prime }$	−21.529	−22.432	−26.833
4.89	1009	$-20^{\circ }25^{\prime }31^{\prime \prime }$	$-21^{\circ }30^{\prime }26^{\prime \prime }$	$-24^{\circ }37^{\prime }27^{\prime \prime }$	−20.425	−21.507	−24.624
4.72	973	$-18^{\circ }17^{\prime }28^{\prime \prime }$	$-20^{\circ }14^{\prime }49^{\prime \prime }$	$-21^{\circ }16^{\prime }06^{\prime \prime }$	−18.291	−20.247	−21.268
4.57	943	$-16^{\circ }25^{\prime }09^{\prime \prime }$	$-19^{\circ }38^{\prime }02^{\prime \prime }$	$-18^{\circ }21^{\prime }17^{\prime \prime }$	−16.419	−19.634	−18.355
4.30	888	$-12^{\circ }49^{\prime }02^{\prime \prime }$	$-16^{\circ }35^{\prime }45^{\prime \prime }$	$-14^{\circ }48^{\prime }16^{\prime \prime }$	−12.817	−16.596	−14.804
4.11	849	$-09^{\circ }25^{\prime }47^{\prime \prime }$	$-13^{\circ }00^{\prime }30^{\prime \prime }$	$-12^{\circ }35^{\prime }17^{\prime \prime }$	−9.430	−13.008	−12.588
3.90	807	$-08^{\circ }08^{\prime }18^{\prime \prime }$	$-09^{\circ }18^{\prime }30^{\prime \prime }$	$-10^{\circ }18^{\prime }45^{\prime \prime }$	−8.138	−9.308	−10.313
3.69	763	$-06^{\circ }10^{\prime }26^{\prime \prime }$	$-07^{\circ }34^{\prime }36^{\prime \prime }$	$-08^{\circ }12^{\prime }08^{\prime \prime }$	−6.174	−7.577	−8.202
3.52	727	$-04^{\circ }06^{\prime }50^{\prime \prime }$	$-04^{\circ }48^{\prime }52^{\prime \prime }$	$-06^{\circ }38^{\prime }18^{\prime \prime }$	−4.114	−4.814	−6.638
3.33	689	$-02^{\circ }33^{\prime }37^{\prime \prime }$	$-03^{\circ }00^{\prime }43^{\prime \prime }$	$-03^{\circ }53^{\prime }42^{\prime \prime }$	−2.560	−3.012	−3.895
3.13	648	$-00^{\circ }25^{\prime }51^{\prime \prime }$	$-00^{\circ }42^{\prime }41^{\prime \prime }$	$-00^{\circ }55^{\prime }07^{\prime \prime }$	−0.431	−0.711	−0.919
2.91	600	$02^{\circ }25^{\prime }05^{\prime \prime }$	$02^{\circ }41^{\prime }30^{\prime \prime }$	$01^{\circ }28^{\prime }15^{\prime \prime }$	2.418	2.692	1.471
2.68	554	$05^{\circ }48^{\prime }59^{\prime \prime }$	$05^{\circ }17^{\prime }14^{\prime \prime }$	$03^{\circ }49^{\prime }32^{\prime \prime }$	5.816	5.287	3.826
2.48	514	$08^{\circ }48^{\prime }29^{\prime \prime }$	$09^{\circ }16^{\prime }13^{\prime \prime }$	$08^{\circ }05^{\prime }43^{\prime \prime }$	8.808	9.270	8.095
2.30	476	$10^{\circ }17^{\prime }37^{\prime \prime }$	$12^{\circ }15^{\prime }32^{\prime \prime }$	$11^{\circ }53^{\prime }16^{\prime \prime }$	10.294	12.259	11.888
2.11	436	$12^{\circ }44^{\prime }41^{\prime \prime }$	$15^{\circ }58^{\prime }38^{\prime \prime }$	$14^{\circ }15^{\prime }42^{\prime \prime }$	12.745	15.977	14.262
1.90	392	$14^{\circ }15^{\prime }27^{\prime \prime }$	$18^{\circ }05^{\prime }35^{\prime \prime }$	$17^{\circ }08^{\prime }36^{\prime \prime }$	14.258	18.093	17.143
1.69	351	$16^{\circ }07^{\prime }11^{\prime \prime }$	$20^{\circ }18^{\prime }06^{\prime \prime }$	$19^{\circ }17^{\prime }18^{\prime \prime }$	16.120	20.302	19.289
1.48	307	$19^{\circ }38^{\prime }57^{\prime \prime }$	$24^{\circ }21^{\prime }43^{\prime \prime }$	$23^{\circ }02^{\prime }30^{\prime \prime }$	19.649	24.362	23.042
1.27	264	$21^{\circ }44^{\prime }12^{\prime \prime }$	$26^{\circ }39^{\prime }34^{\prime \prime }$	$25^{\circ }57^{\prime }8^{\prime \prime }$	21.737	26.660	25.952
1.14	236	$22^{\circ }55^{\prime }3^{\prime \prime }$	$29^{\circ }45^{\prime }35^{\prime \prime }$	$28^{\circ }58^{\prime }21^{\prime \prime }$	22.918	29.760	28.973
1.01	210	$23^{\circ }34^{\prime }55^{\prime \prime }$	$31^{\circ }25^{\prime }47^{\prime \prime }$	$30^{\circ }41^{\prime }24^{\prime \prime }$	23.582	31.430	30.690

**Table 6:** Polynomial Regression Analysis of Arduino Digital Reading and Motor Angle.

Model	No. of Params.	MSE	R-Squared	Prob (F-Statistic)
Polynomial (Degree 2)	3	0.3505	0.9983	4.38e-27
Polynomial (Degree 3)	4	0.3487	0.9983	3.59e-25
Polynomial (Degree 4)	5	0.2505	0.9988	1.42e-24
Polynomial (Degree 5)	6	0.3534	0.9983	2.65e-23
Polynomial (Degree 6)	7	2.0122	0.9904	6.94e-17
Polynomial (Degree 7)	8	10.6927	0.9487	9.78e-11
Polynomial (Degree 8)	9	31.1236	0.8508	1.20e-07
Polynomial (Degree 9)	10	104.4090	0.4995	5.15e-03
Polynomial (Degree 10)	11	114.8343	0.4495	1.16e-02

**Table 7:** Polynomial Regression Analysis of Motor Angle and Left Steering Angle.

Model	No. of Params.	MSE	R-Squared	Prob (F-Statistic)
Polynomial (Degree 1)	2	2.0355	0.9933	3.25e-23
Polynomial (Degree 2)	3	0.7265	0.9976	1.26e-25
Polynomial (Degree 3)	4	0.6860	0.9977	5.42e-24
Polynomial (Degree 4)	5	0.5280	0.9983	3.32e-23
Polynomial (Degree 5)	6	0.4850	0.9984	8.97e-22
Polynomial (Degree 6)	7	0.4761	0.9984	3.72e-20
Polynomial (Degree 7)	8	0.1315	0.9996	1.93e-22
Polynomial (Degree 8)	9	0.1273	0.9996	1.12e-20
Polynomial (Degree 9)	10	0.1254	0.9996	6.72e-19
Polynomial (Degree 10)	11	0.1244	0.9996	3.84e-17

**Table 8:** Polynomial Regression Analysis of Motor Angle and Right Steering Angle.

Model	No. of Params.	MSE	R-Squared	Prob (F-Statistic)
Polynomial (Degree 1)	2	1.5021	0.9951	1.40e-24
Polynomial (Degree 2)	3	0.8670	0.9972	6.14e-25
Polynomial (Degree 3)	4	0.6272	0.9979	2.21e-24
Polynomial (Degree 4)	5	0.5551	0.9982	4.65e-23
Polynomial (Degree 5)	6	0.4837	0.9984	8.09e-22
Polynomial (Degree 6)	7	0.4829	0.9984	3.84e-20
Polynomial (Degree 7)	8	0.3185	0.9990	8.81e-20
Polynomial (Degree 8)	9	0.2286	0.9993	4.72e-19
Polynomial (Degree 9)	10	0.1987	0.9994	1.00e-17
Polynomial (Degree 10)	11	0.1742	0.9994	2.31e-16

**Table 9:** PID parameters for each algorithm in steering control of the scooter.

Algorithm Name	$K_{p}$	$K_{i}$	$K_{d}$
Manual Tuning	5	30	0.15
Ziegler–Nichols	18	31.92	2.54
HHO Algorithm	33.1505	67.012	0.9064
DHHO Algorithm	14.1019	86.4520	1.0117

**Table 10:** PID Tuning results and performance metrics in steering control.

Algorithm	Rise Time	Peak Time	Settling Time	Max Overshoot	Steady-State Error
Manual	0.0388	0.0800	0.3781	9.0890	0.0000
ZN	0.0162	0.0500	0.7854	3.3427	0.0000
HHO	0.0169	0.0400	0.0745	8.3082	0.0000
DHHO	0.0224	0.6600	0.0404	0.0000	0.0000

## Data Availability

No datasets were used during the current study. The MATLAB source code and the result files for the proposed algorithm are available in the Supplementary Materials File and at the following GitHub repository https://github.com/MohamedRedaMu/DHHO (accessed on 12 July 2024).

## Supplementary Materials

The following supporting information can be downloaded at: https://www.mdpi.com/article/10.3390/s24144650/s1, Algorithm S1: The Harris Hawks Optimization (HHO) algorithm. Figure S1: The flowchart of the proposed DHHO algorithm. Figure S2: Population diversity plots (variance and SD) between HHO and DHHO exploration methods for dimensions (10–100). Figure S3: Box and violin plots for all the 10-Dim CEC2020/2021 functions. Figure S4: Box and violin plots for all the 20-Dim CEC2020/2021 functions. Figure S5: Steering angle mapping readings. Figure S6: Regression results for the relationship between the motor rotation angle and the encoder digital reading. Figure S7: Regression results for the relationship between the motor rotation and left steering angles. Figure S8: Regression results for the relationship between the motor rotation and right steering angles. Table S1: Performance comparison of the DHHO and traditional HHO algorithms on CEC2021 benchmark functions.
