# Supplementary material for: Optimizing the Steering of Driverless Personal Mobility Pods with a Novel Differential Harris Hawks Optimization Algorithm (DHHO) and Encoder Modeling
Source: Sensors (Basel). 2024 Jul 17;24(14):4650. doi: 10.3390/s24144650 (PMC11281304; doi:10.3390/s24144650)
Supplement: Supplementary file 1 [file sensors-24-04650-s001.zip › sensors-3031786-supplementary.pdf]

# Supplementary Materials

**Algorithm S1** Harris Hawks Optimization (HHO) Algorithm

Initialize the population $X_i$, $i = 1, 2, \ldots, N$
Evaluate the fitness of each hawk
Initialize the best solution found so far as the rabbit's location $R$
**for** $t = 1$ to $T$ **do** ▷ T is the maximum number of iterations
    **for** each hawk $i$ **do**
        Update energy $E$ of the rabbit from Eq.(1)
        **if** Rabbit's energy is high **then** ▷ Exploration phase
            Update hawk's position based on Eq.(2).
        **else** ▷ Exploitation phase
            Update hawk's position based on Eq.(3)
        **end if**
        Evaluate fitness of hawk $i$
        **if** Fitness of hawk $i$ is better than $R$ **then**
            Update $R$ with hawk $i$'s position
        **end if**
    **end for**
    Record the best solution found so far
**end for**
**return** Best solution found $R$

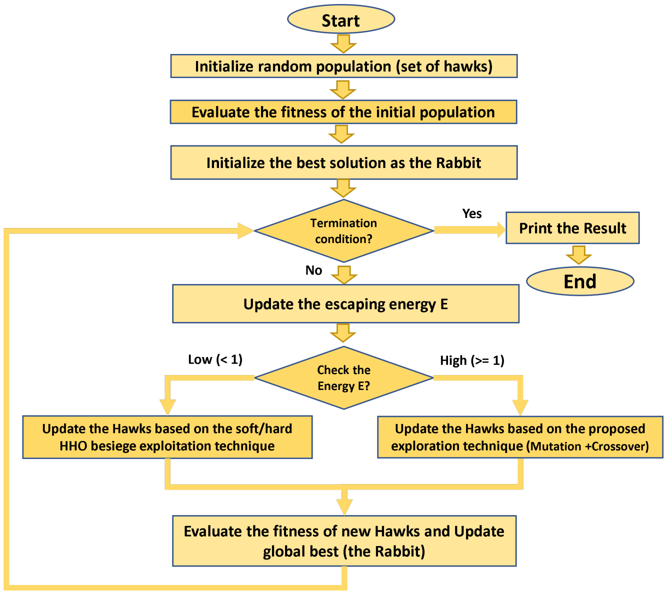


Figure S1: The flowchart of the proposed DHHO algorithm.

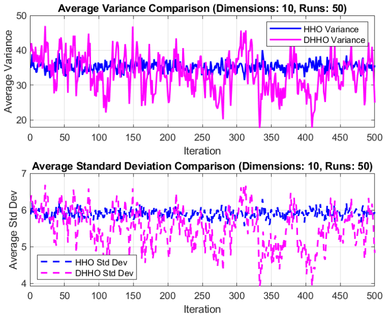


(a) 10 Dimensions.

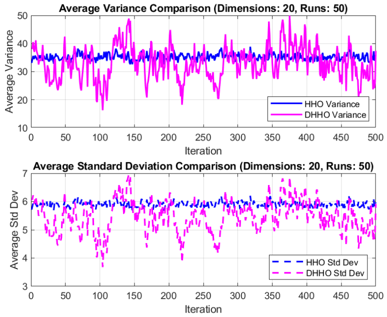


(b) 20 Dimensions.

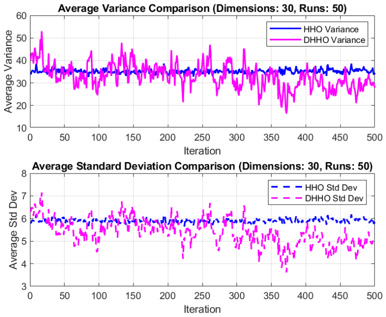


(c) 30 Dimensions.

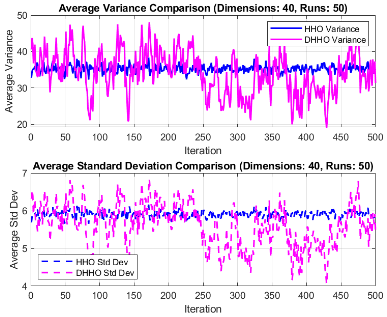


(d) 40 Dimensions.

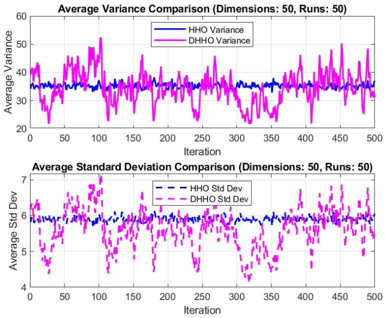


(e) 50 Dimensions.

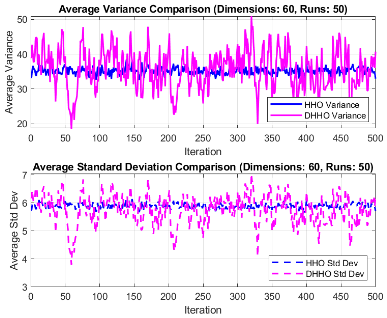


(f) 60 Dimensions.

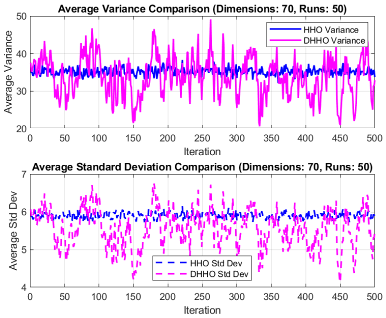


(g) 70 Dimensions.

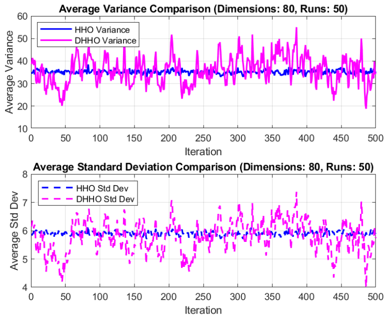


(h) 80 Dimensions.

Figure S2: Population diversity plots (variance and SD) between HHO and DHHO exploration methods.

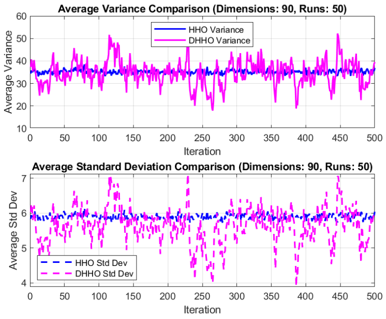


(i) 90 Dimensions.

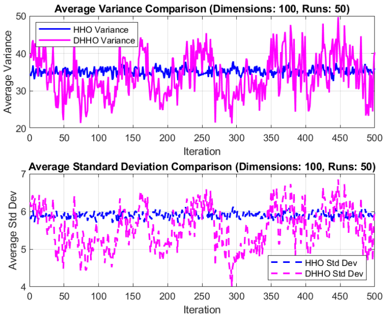


(j) 100 Dimensions.

Fig. S2(Cont.) Population diversity plots (variance and SD) between HHO and DHHO exploration methods.

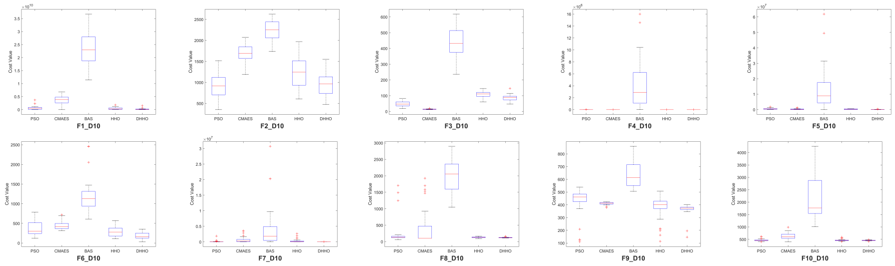


(a) Box plots.

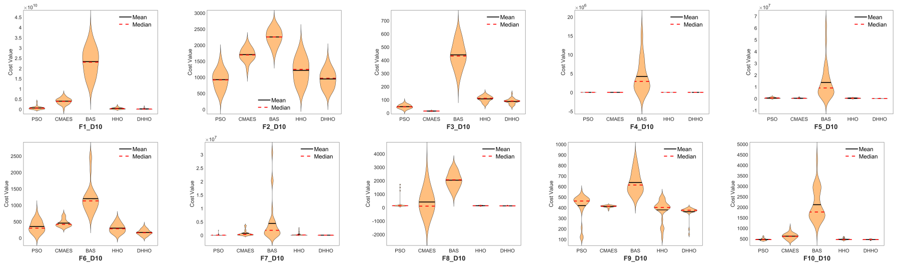


(b) Violin plots.

Figure S3: Box and violin plots for all the 10-Dim CEC2020/2021 functions for all the algorithms in 30 runs.

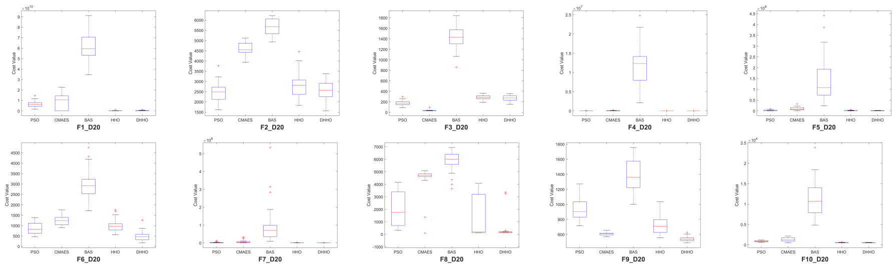


(a) Box plots.

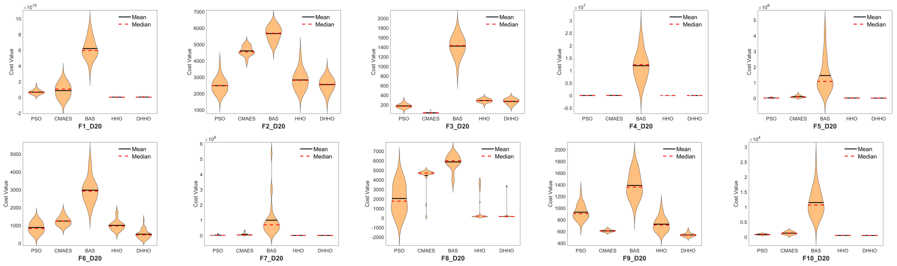


(b) Violin plots.

Figure S4: Box and violin plots for all the 20-Dim CEC2020/2021 functions for all the algorithms in 30 runs.

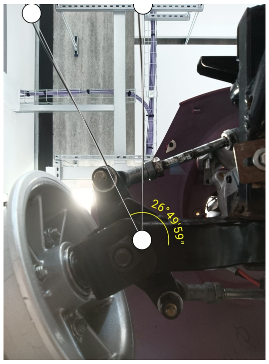


(a) Right Angle - 4.93V.

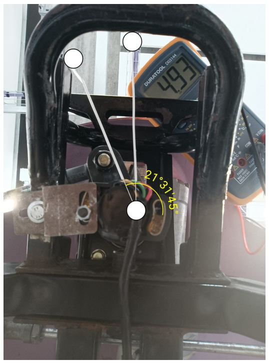


(b) Motor Angle - 4.93V.

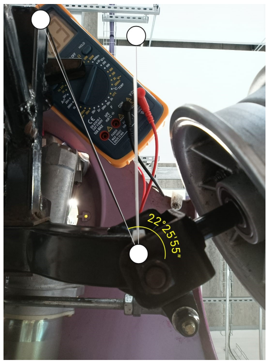


(c) Left Angle - 4.93V.

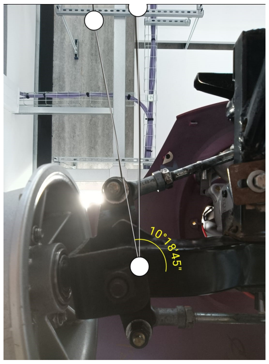


(d) Right Angle - 3.90V.

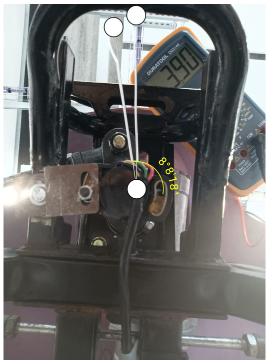


(e) Motor Angle - 3.90V.

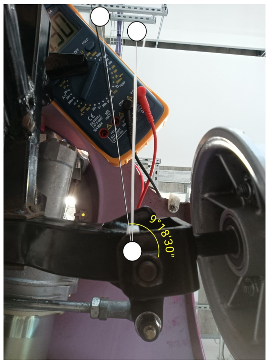


(f) Left Angle - 3.90V.

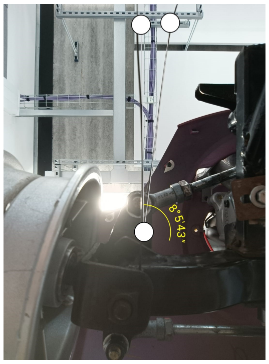


(g) Right Angle - 2.49V.

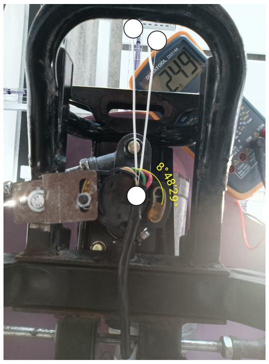


(h) Motor Angle - 2.49V.

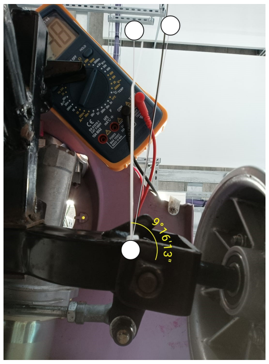


(i) Left Angle - 2.49V.

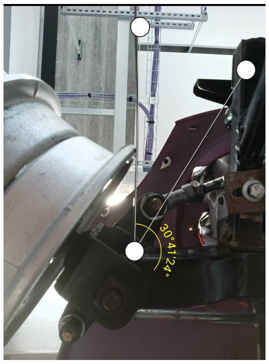


(j) Right Angle - 1.01V.

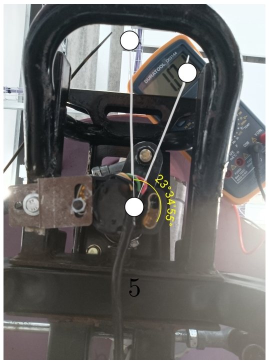


(k) Motor Angle - 1.01V.

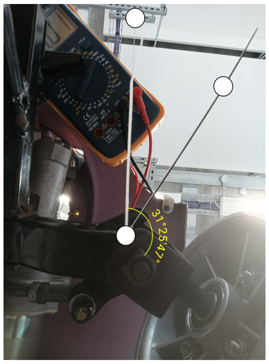


(l) Left Angle - 1.01V.

Figure S5: Steering angle mapping readings.

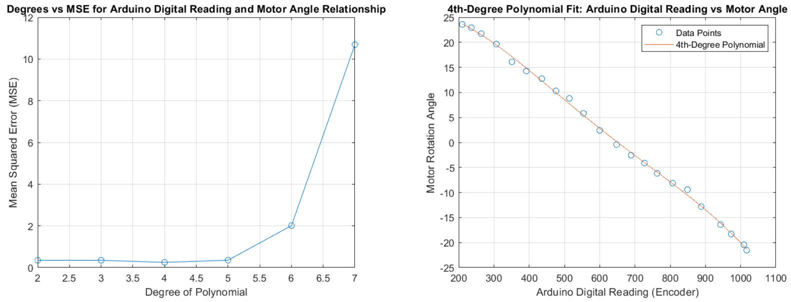


(a) MSE vs the polynomial degree. (b) Graphical relationship (4th deg.).

Figure S6: Regression results for the relationship between the motor rotation angle and the encoder digital reading.

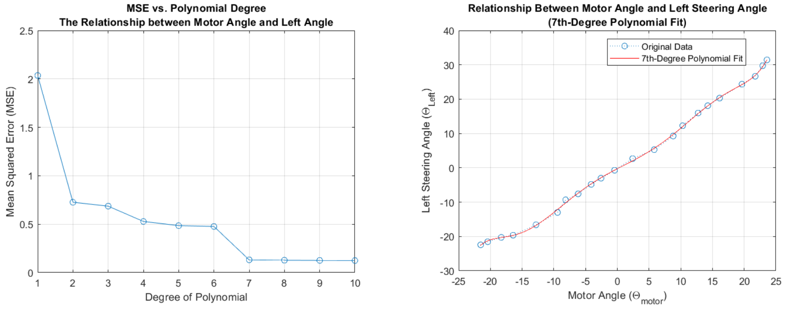


(a) MSE vs polynomial degree. (b) Graphical relationship (7th deg.).

Figure S7: Regression results for the relationship between the motor rotation and left steering angles.

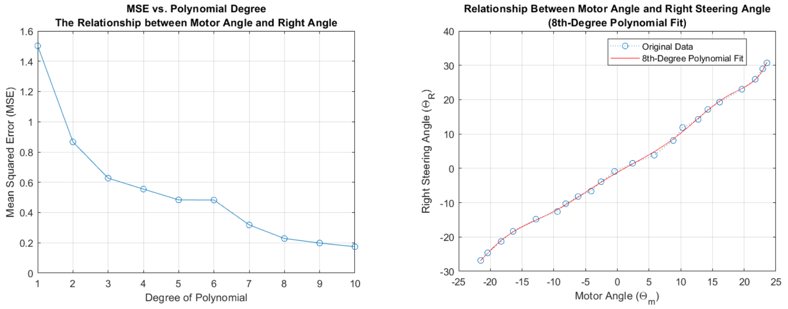


(a) MSE vs the polynomial degree. (b) Graphical relationship (8th deg.).

Figure S8: Regression results for the relationship between the motor rotation and right steering angles.

Table S1 shows each controller's steering angle response at 20 timestamps for different steering angles.

Table S1: Data collected during measuring the transient response for each controller (Manual, ZN, HHO, and the proposed DHHO) at different steering angles.

| Ref. | Alg. | 0 | 0.0028 | 0.0063 | 0.0108 | 0.0165 | 0.0238 | 0.0331 | 0.0450 | 0.0601 | 0.0794 | 0.1039 | 0.1352 | 0.1750 | 0.2258 | 0.2905 | 0.3730 | 0.4781 | 0.6120 | 0.7826 | 1.0000 |
|---|---|---|---|---|---|---|---|---|---|---|---|---|---|---|---|---|---|---|---|---|---|
| -21 | Manual | 0 | -1.63 | -3.71 | -6.30 | -9.23 | -12.61 | -16.19 | -19.50 | -22.03 | -22.90 | -22.02 | -20.21 | -19.10 | -19.49 | -20.29 | -20.57 | -20.76 | -20.90 | -20.96 | -20.99 |
| | ZN | 0 | -4.25 | -9.67 | -15.73 | -18.40 | -20.54 | -21.47 | -21.70 | -21.62 | -21.42 | -21.19 | -20.94 | -20.71 | -20.51 | -20.36 | -20.30 | -20.32 | -20.43 | -20.58 | -20.73 |
| | HHO | 0 | -3.78 | -8.59 | -14.13 | -17.70 | -20.77 | -22.39 | -22.62 | -22.03 | -21.25 | -20.77 | -20.62 | -20.63 | -20.67 | -20.71 | -20.76 | -20.81 | -20.85 | -20.90 | -20.94 |
| | DHHO | 0 | -3.70 | -8.42 | -13.75 | -16.52 | -18.87 | -20.14 | -20.68 | -20.86 | -20.88 | -20.86 | -20.86 | -20.87 | -20.89 | -20.93 | -20.97 | -20.99 | -21.00 | -21.00 | -21.00 |
| -18 | Manual | 0 | -1.40 | -3.18 | -5.40 | -7.91 | -10.81 | -13.88 | -16.71 | -18.89 | -19.63 | -18.87 | -17.33 | -16.37 | -16.70 | -17.39 | -17.63 | -17.79 | -17.91 | -17.97 | -17.99 |
| | ZN | 0 | -3.65 | -8.29 | -13.48 | -15.77 | -17.60 | -18.40 | -18.60 | -18.53 | -18.36 | -18.16 | -17.95 | -17.75 | -17.58 | -17.45 | -17.40 | -17.42 | -17.51 | -17.64 | -17.77 |
| | HHO | 0 | -3.24 | -7.36 | -12.11 | -15.17 | -17.80 | -19.19 | -19.39 | -18.88 | -18.21 | -17.80 | -17.68 | -17.68 | -17.72 | -17.75 | -17.79 | -17.83 | -17.87 | -17.91 | -17.94 |
| | DHHO | 0 | -3.17 | -7.21 | -11.78 | -14.16 | -16.18 | -17.27 | -17.73 | -17.88 | -17.89 | -17.88 | -17.88 | -17.89 | -17.91 | -17.94 | -17.97 | -17.99 | -18.00 | -18.00 | -18.00 |
| -15 | Manual | 0 | -1.17 | -2.65 | -4.50 | -6.59 | -9.01 | -11.56 | -13.93 | -15.74 | -16.36 | -15.73 | -14.44 | -13.64 | -13.92 | -14.49 | -14.69 | -14.83 | -14.93 | -14.97 | -14.99 |
| | ZN | 0 | -3.04 | -6.91 | -11.23 | -13.14 | -14.67 | -15.34 | -15.50 | -15.44 | -15.30 | -15.13 | -14.96 | -14.79 | -14.65 | -14.54 | -14.50 | -14.52 | -14.59 | -14.70 | -14.81 |
| | HHO | 0 | -2.70 | -6.13 | -10.09 | -12.64 | -14.83 | -15.99 | -16.16 | -15.74 | -15.18 | -14.83 | -14.73 | -14.74 | -14.77 | -14.80 | -14.83 | -14.86 | -14.90 | -14.93 | -14.95 |
| | DHHO | 0 | -2.64 | -6.01 | -9.82 | -11.80 | -13.48 | -14.39 | -14.77 | -14.90 | -14.91 | -14.90 | -14.90 | -14.90 | -14.92 | -14.95 | -14.98 | -14.99 | -15.00 | -15.00 | -15.00 |
| -13 | Manual | 0 | -1.01 | -2.30 | -3.90 | -5.71 | -7.81 | -10.02 | -12.07 | -13.64 | -14.17 | -13.63 | -12.51 | -11.82 | -12.06 | -12.56 | -12.73 | -12.85 | -12.94 | -12.98 | -12.99 |
| | ZN | 0 | -2.63 | -5.99 | -9.73 | -11.39 | -12.71 | -13.29 | -13.43 | -13.38 | -13.26 | -13.12 | -12.96 | -12.82 | -12.69 | -12.61 | -12.57 | -12.58 | -12.65 | -12.74 | -12.84 |
| | HHO | 0 | -2.34 | -5.32 | -8.75 | -10.96 | -12.86 | -13.86 | -14.01 | -13.64 | -13.15 | -12.86 | -12.77 | -12.77 | -12.80 | -12.82 | -12.85 | -12.88 | -12.91 | -12.94 | -12.96 |
| | DHHO | 0 | -2.29 | -5.21 | -8.51 | -10.23 | -11.68 | -12.47 | -12.80 | -12.91 | -12.92 | -12.92 | -12.91 | -12.92 | -12.93 | -12.96 | -12.98 | -13.00 | -13.00 | -13.00 | -13.00 |
| -10 | Manual | 0 | -0.78 | -1.77 | -3.00 | -4.40 | -6.01 | -7.71 | -9.28 | -10.49 | -10.90 | -10.48 | -9.63 | -9.09 | -9.28 | -9.66 | -9.80 | -9.88 | -9.95 | -9.98 | -10.00 |
| | ZN | 0 | -2.03 | -4.61 | -7.49 | -8.76 | -9.78 | -10.22 | -10.33 | -10.30 | -10.20 | -10.09 | -9.97 | -9.86 | -9.77 | -9.70 | -9.67 | -9.68 | -9.73 | -9.80 | -9.87 |
| | HHO | 0 | -1.80 | -4.09 | -6.73 | -8.43 | -9.89 | -10.66 | -10.77 | -10.49 | -10.12 | -9.89 | -9.82 | -9.82 | -9.84 | -9.86 | -9.89 | -9.91 | -9.93 | -9.95 | -9.97 |
| | DHHO | 0 | -1.76 | -4.01 | -6.55 | -7.87 | -8.99 | -9.59 | -9.85 | -9.93 | -9.94 | -9.94 | -9.93 | -9.94 | -9.95 | -9.97 | -9.98 | -10.00 | -10.00 | -10.00 | -10.00 |
| -7 | Manual | 0 | -0.54 | -1.24 | -2.10 | -3.08 | -4.20 | -5.40 | -6.50 | -7.34 | -7.63 | -7.34 | -6.74 | -6.37 | -6.50 | -6.76 | -6.86 | -6.92 | -6.97 | -6.99 | -7.00 |
| | ZN | 0 | -1.42 | -3.22 | -5.24 | -6.13 | -6.85 | -7.16 | -7.23 | -7.21 | -7.14 | -7.06 | -6.98 | -6.90 | -6.84 | -6.79 | -6.77 | -6.77 | -6.81 | -6.86 | -6.91 |
| | HHO | 0 | -1.26 | -2.86 | -4.71 | -5.90 | -6.92 | -7.46 | -7.54 | -7.34 | -7.08 | -6.92 | -6.87 | -6.88 | -6.89 | -6.90 | -6.92 | -6.94 | -6.95 | -6.97 | -6.98 |
| | DHHO | 0 | -1.23 | -2.81 | -4.58 | -5.51 | -6.29 | -6.71 | -6.89 | -6.95 | -6.96 | -6.95 | -6.95 | -6.96 | -6.96 | -6.98 | -6.99 | -7.00 | -7.00 | -7.00 | -7.00 |
| -4 | Manual | 0 | -0.31 | -0.71 | -1.20 | -1.76 | -2.40 | -3.08 | -3.71 | -4.20 | -4.36 | -4.19 | -3.85 | -3.64 | -3.71 | -3.86 | -3.92 | -3.95 | -3.98 | -3.99 | -4.00 |
| | ZN | 0 | -0.81 | -1.84 | -3.00 | -3.50 | -3.91 | -4.09 | -4.13 | -4.12 | -4.08 | -4.04 | -3.99 | -3.94 | -3.91 | -3.88 | -3.87 | -3.87 | -3.89 | -3.92 | -3.95 |
| | HHO | 0 | -0.72 | -1.64 | -2.69 | -3.37 | -3.96 | -4.26 | -4.31 | -4.20 | -4.05 | -3.96 | -3.93 | -3.93 | -3.94 | -3.95 | -3.95 | -3.96 | -3.97 | -3.98 | -3.99 |
| | DHHO | 0 | -0.70 | -1.60 | -2.62 | -3.15 | -3.59 | -3.84 | -3.94 | -3.97 | -3.98 | -3.97 | -3.97 | -3.97 | -3.98 | -3.99 | -3.99 | -4.00 | -4.00 | -4.00 | -4.00 |
| -1 | Manual | 0 | -0.08 | -0.18 | -0.30 | -0.44 | -0.60 | -0.77 | -0.93 | -1.05 | -1.09 | -1.05 | -0.96 | -0.91 | -0.93 | -0.97 | -0.98 | -0.99 | -1.00 | -1.00 | -1.00 |
| | ZN | 0 | -0.20 | -0.46 | -0.75 | -0.88 | -0.98 | -1.02 | -1.03 | -1.03 | -1.02 | -1.01 | -1.00 | -0.99 | -0.98 | -0.97 | -0.97 | -0.97 | -0.97 | -0.98 | -0.99 |
| | HHO | 0 | -0.18 | -0.41 | -0.67 | -0.84 | -0.99 | -1.07 | -1.08 | -1.05 | -1.01 | -0.99 | -0.98 | -0.98 | -0.98 | -0.99 | -0.99 | -0.99 | -0.99 | -1.00 | -1.00 |
| | DHHO | 0 | -0.18 | -0.40 | -0.65 | -0.79 | -0.90 | -0.96 | -0.98 | -0.99 | -0.99 | -0.99 | -0.99 | -0.99 | -0.99 | -1.00 | -1.00 | -1.00 | -1.00 | -1.00 | -1.00 |
| 1 | Manual | 0 | 0.08 | 0.18 | 0.30 | 0.44 | 0.60 | 0.77 | 0.93 | 1.05 | 1.09 | 1.05 | 0.96 | 0.91 | 0.93 | 0.97 | 0.98 | 0.99 | 1.00 | 1.00 | 1.00 |
| | ZN | 0 | 0.20 | 0.46 | 0.75 | 0.88 | 0.98 | 1.02 | 1.03 | 1.03 | 1.02 | 1.01 | 1.00 | 0.99 | 0.98 | 0.97 | 0.97 | 0.97 | 0.97 | 0.98 | 0.99 |
| | HHO | 0 | 0.18 | 0.41 | 0.67 | 0.84 | 0.99 | 1.07 | 1.08 | 1.05 | 1.01 | 0.99 | 0.98 | 0.98 | 0.98 | 0.99 | 0.99 | 0.99 | 0.99 | 1.00 | 1.00 |
| | DHHO | 0 | 0.18 | 0.40 | 0.65 | 0.79 | 0.90 | 0.96 | 0.98 | 0.99 | 0.99 | 0.99 | 0.99 | 0.99 | 0.99 | 1.00 | 1.00 | 1.00 | 1.00 | 1.00 | 1.00 |
| 4 | Manual | 0 | 0.31 | 0.71 | 1.20 | 1.76 | 2.40 | 3.08 | 3.71 | 4.20 | 4.36 | 4.19 | 3.85 | 3.64 | 3.71 | 3.86 | 3.92 | 3.95 | 3.98 | 3.99 | 4.00 |
| | ZN | 0 | 0.81 | 1.84 | 3.00 | 3.50 | 3.91 | 4.09 | 4.13 | 4.12 | 4.08 | 4.04 | 3.99 | 3.94 | 3.91 | 3.88 | 3.87 | 3.87 | 3.89 | 3.92 | 3.95 |
| | HHO | 0 | 0.72 | 1.64 | 2.69 | 3.37 | 3.96 | 4.26 | 4.31 | 4.20 | 4.05 | 3.96 | 3.93 | 3.93 | 3.94 | 3.95 | 3.95 | 3.96 | 3.97 | 3.98 | 3.99 |
| | DHHO | 0 | 0.70 | 1.60 | 2.62 | 3.15 | 3.59 | 3.84 | 3.94 | 3.97 | 3.98 | 3.97 | 3.97 | 3.97 | 3.98 | 3.99 | 3.99 | 4.00 | 4.00 | 4.00 | 4.00 |
| 7 | Manual | 0 | 0.54 | 1.24 | 2.10 | 3.08 | 4.20 | 5.40 | 6.50 | 7.34 | 7.63 | 7.34 | 6.74 | 6.37 | 6.50 | 6.76 | 6.86 | 6.92 | 6.97 | 6.99 | 7.00 |
| | ZN | 0 | 1.42 | 3.22 | 5.24 | 6.13 | 6.85 | 7.16 | 7.23 | 7.21 | 7.14 | 7.06 | 6.98 | 6.90 | 6.84 | 6.79 | 6.77 | 6.77 | 6.81 | 6.86 | 6.91 |
| | HHO | 0 | 1.26 | 2.86 | 4.71 | 5.90 | 6.92 | 7.46 | 7.54 | 7.34 | 7.08 | 6.92 | 6.87 | 6.88 | 6.89 | 6.90 | 6.92 | 6.94 | 6.95 | 6.97 | 6.98 |
| | DHHO | 0 | 1.23 | 2.81 | 4.58 | 5.51 | 6.29 | 6.71 | 6.89 | 6.95 | 6.96 | 6.95 | 6.95 | 6.96 | 6.96 | 6.98 | 6.99 | 7.00 | 7.00 | 7.00 | 7.00 |
| 10 | Manual | 0 | 0.78 | 1.77 | 3.00 | 4.40 | 6.01 | 7.71 | 9.28 | 10.49 | 10.90 | 10.48 | 9.63 | 9.09 | 9.28 | 9.66 | 9.80 | 9.88 | 9.95 | 9.98 | 10.00 |
| | ZN | 0 | 2.03 | 4.61 | 7.49 | 8.76 | 9.78 | 10.22 | 10.33 | 10.30 | 10.20 | 10.09 | 9.97 | 9.86 | 9.77 | 9.70 | 9.67 | 9.68 | 9.73 | 9.80 | 9.87 |
| | HHO | 0 | 1.80 | 4.09 | 6.73 | 8.43 | 9.89 | 10.66 | 10.77 | 10.49 | 10.12 | 9.89 | 9.82 | 9.82 | 9.84 | 9.86 | 9.89 | 9.91 | 9.93 | 9.95 | 9.97 |
| | DHHO | 0 | 1.76 | 4.01 | 6.55 | 7.87 | 8.99 | 9.59 | 9.85 | 9.93 | 9.94 | 9.94 | 9.93 | 9.94 | 9.95 | 9.97 | 9.98 | 10.00 | 10.00 | 10.00 | 10.00 |
| 13 | Manual | 0 | 1.01 | 2.30 | 3.90 | 5.71 | 7.81 | 10.02 | 12.07 | 13.64 | 14.17 | 13.63 | 12.51 | 11.82 | 12.06 | 12.56 | 12.73 | 12.85 | 12.94 | 12.98 | 12.99 |
| | ZN | 0 | 2.63 | 5.99 | 9.73 | 11.39 | 12.71 | 13.29 | 13.43 | 13.38 | 13.26 | 13.12 | 12.96 | 12.82 | 12.69 | 12.61 | 12.57 | 12.58 | 12.65 | 12.74 | 12.84 |
| | HHO | 0 | 2.34 | 5.32 | 8.75 | 10.96 | 12.86 | 13.86 | 14.01 | 13.64 | 13.15 | 12.86 | 12.77 | 12.77 | 12.80 | 12.82 | 12.85 | 12.88 | 12.91 | 12.94 | 12.96 |
| | DHHO | 0 | 2.29 | 5.21 | 8.51 | 10.23 | 11.68 | 12.47 | 12.80 | 12.91 | 12.92 | 12.92 | 12.91 | 12.92 | 12.93 | 12.96 | 12.98 | 13.00 | 13.00 | 13.00 | 13.00 |
| 15 | Manual | 0 | 1.17 | 2.65 | 4.50 | 6.59 | 9.01 | 11.56 | 13.93 | 15.74 | 16.36 | 15.73 | 14.44 | 13.64 | 13.92 | 14.49 | 14.69 | 14.83 | 14.93 | 14.97 | 14.99 |
| | ZN | 0 | 3.04 | 6.91 | 11.23 | 13.14 | 14.67 | 15.34 | 15.50 | 15.44 | 15.30 | 15.13 | 14.96 | 14.79 | 14.65 | 14.54 | 14.50 | 14.52 | 14.59 | 14.70 | 14.81 |
| | HHO | 0 | 2.70 | 6.13 | 10.09 | 12.64 | 14.83 | 15.99 | 16.16 | 15.74 | 15.18 | 14.83 | 14.73 | 14.74 | 14.77 | 14.80 | 14.83 | 14.86 | 14.90 | 14.93 | 14.95 |
| | DHHO | 0 | 2.64 | 6.01 | 9.82 | 11.80 | 13.48 | 14.39 | 14.77 | 14.90 | 14.91 | 14.90 | 14.90 | 14.90 | 14.92 | 14.95 | 14.98 | 14.99 | 15.00 | 15.00 | 15.00 |
| 18 | Manual | 0 | 1.40 | 3.18 | 5.40 | 7.91 | 10.81 | 13.88 | 16.71 | 18.89 | 19.63 | 18.87 | 17.33 | 16.37 | 16.70 | 17.39 | 17.63 | 17.79 | 17.91 | 17.97 | 17.99 |
| | ZN | 0 | 3.65 | 8.29 | 13.48 | 15.77 | 17.60 | 18.40 | 18.60 | 18.53 | 18.36 | 18.16 | 17.95 | 17.75 | 17.58 | 17.45 | 17.40 | 17.42 | 17.51 | 17.64 | 17.77 |
| | HHO | 0 | 3.24 | 7.36 | 12.11 | 15.17 | 17.80 | 19.19 | 19.39 | 18.88 | 18.21 | 17.80 | 17.68 | 17.68 | 17.72 | 17.75 | 17.79 | 17.83 | 17.87 | 17.91 | 17.94 |
| | DHHO | 0 | 3.17 | 7.21 | 11.78 | 14.16 | 16.18 | 17.27 | 17.73 | 17.88 | 17.89 | 17.88 | 17.88 | 17.89 | 17.91 | 17.94 | 17.97 | 17.99 | 18.00 | 18.00 | 18.00 |
| 21 | Manual | 0 | 1.63 | 3.71 | 6.30 | 9.23 | 12.61 | 16.19 | 19.50 | 22.03 | 22.90 | 22.02 | 20.21 | 19.10 | 19.49 | 20.29 | 20.57 | 20.76 | 20.90 | 20.96 | 20.99 |
| | ZN | 0 | 4.25 | 9.67 | 15.73 | 18.40 | 20.54 | 21.47 | 21.70 | 21.62 | 21.42 | 21.19 | 20.94 | 20.71 | 20.51 | 20.36 | 20.30 | 20.32 | 20.43 | 20.58 | 20.73 |
| | HHO | 0 | 3.78 | 8.59 | 14.13 | 17.70 | 20.77 | 22.39 | 22.62 | 22.03 | 21.25 | 20.77 | 20.62 | 20.63 | 20.67 | 20.71 | 20.76 | 20.81 | 20.85 | 20.90 | 20.94 |
| | DHHO | 0 | 3.70 | 8.42 | 13.75 | 16.52 | 18.87 | 20.14 | 20.68 | 20.86 | 20.88 | 20.86 | 20.86 | 20.87 | 20.89 | 20.93 | 20.97 | 20.99 | 21.00 | 21.00 | 21.00 |
